# Effects of Brain-Computer Interface-Controlled Hand Robot Training on Post-Stroke Recovery of Upper Limb Motor Functions: A Meta-Analysis of Dose-Matched Randomized Controlled Trials

**DOI:** 10.3390/brainsci16060552

**Published:** 2026-05-22

**Authors:** Song Hu, Fengjiao Wang, Xiaoxue Gao, Yong Zhi, Daehee Kim

**Affiliations:** 1 Graduate School of Physical Education, Pukyong National University, Busan 48513, Republic of Korea; pknuhu04@163.com (S.H.); 19983992687@163.com (X.G.); 2 School of Health Sciences, Universiti Sains Malaysia, Kubang Kerian 16150, Malaysia; wangfengjiao2024@163.com; 3 Department of Industrial Design, Pukyong National University, Busan 48513, Republic of Korea; ghoul1024@gmail.com

**Keywords:** brain-computer interface, hand robot, upper limb function

## Abstract

**Highlights:**

**What are the main findings?**

- BCI-controlled hand robot training improved overall upper-limb motor function and reduced finger flexor spasticity after stroke. Its effect on upper-limb motor function was more evident in subacute stroke patients.
- BCI-controlled hand robot training showed greater effects on proximal upper-limb motor function than on distal upper-limb motor function.

**What are the implications of the main findings?**

- This study provides dose-matched evidence supporting the clinical use of BCI-controlled hand robot training in post-stroke upper-limb rehabilitation.
- Future trials should optimize BCI paradigms and robot types, especially to improve distal hand function and long-term efficacy.

**Abstract:**

**Objective**: To systematically evaluate the rehabilitation effect of brain-computer interface (BCI)-controlled hand robot training on post-stroke motor functions, especially upper limb functions. **Methods**: PubMed, Embase, Web of Science, Cochrane Library, CNKI, SinoMed, WanFang Data, and VIP Database were searched from inception to 13 March 2026. Randomized controlled trials (RCTs) with dose-matched designs were included, where the test group underwent BCI-controlled hand robot training and the control group received either pure hand robot training or routine rehabilitation. Meta-analysis was performed on RevMan 5.4. **Results**: Totally 11 RCTs involving 380 patients were included. Compared with hand robot training alone, BCI-controlled hand robot training significantly improved Fugl-Meyer Assessment for Upper Extremity (FMA-UE) scores (MD = 4.87, 95% CI: 1.04 to 8.69) and FMA-UE proximal scores (MD = 4.44, 95% CI: 0.15 to 8.74), and significantly reduced finger flexor spasticity (MD = −0.44, 95% CI: −0.68 to −0.21), but showed no significant difference in distal upper limb motor function or Action Research Arm Test (ARAT) scores. Compared with routine rehabilitation, BCI-controlled hand robot training significantly improved FMA-UE scores (MD = 6.55, 95% CI: 3.49 to 9.61). **Conclusions**: BCI-controlled hand robot training can effectively improve overall upper limb and proximal motor function after stroke and alleviate finger flexor spasticity, but the evidence for distal hand function and long-term efficacy remains limited.

## 1. Introduction

Stroke, also known as cerebrovascular accident, is an acute cerebrovascular disease caused by the blockage or rupture of cerebral blood vessels. It can cause cerebral tissue injury and corresponding neural dysfunction, and is one major disabling disease worldwide [1]. Upper limb motor dysfunction is a primary complication following stroke [2]. Particularly, impaired hand function has become a core factor affecting the self-care ability of stroke patients [3]. Therefore, restoring upper limb motor function is one major challenge in the field of neurological rehabilitation [2].

The brain-computer interface (BCI) is a neural technology that converts cerebral activity signals into control commands for external devices [4]. It is mainly divided, by the way of acquiring cerebral signals, into invasive and non-invasive types [5]. Currently, the most commonly used BCI systems in stroke rehabilitation are non-invasive systems [6]. Non-invasive BCIs typically use magnetoencephalography (MEG), electroencephalography (EEG), or functional near-infrared spectroscopy (fNIRS) to collect brain signals from the scalp surface [7]. After cerebral signals are amplified, filtered, classified and decoded, the patient’s movement intention is converted into control commands for external devices to regulate prosthetics, robots or electrical stimulation systems, and feedback is provided in visual, auditory or tactile forms [5] to enhance rehabilitation effectiveness [8]. Among them, EEG with high resolution, portability and relatively low cost is the most widely used non-invasive BCI signal acquisition method in stroke rehabilitation research [9]. Most EEG-based BCI systems primarily employ paradigms such as event-related desynchronization (ERD), event-related potential (ERP), and steady-state visual evoked potential (SSVEP) [10]. Among them, motor imagery BCI (MI-BCI) training based on ERD detection is one of the most commonly used paradigms for stroke rehabilitation [7]. Since MI-BCI can identify and decode the patient’s motor intentions, an important application direction in stroke rehabilitation is to integrate it with the robot system to convert neural signals into practical motor output [11]. Robots are applied in stroke rehabilitation primarily to facilitate motor learning and improve the recovery of motor function through repetitive task-oriented training [12,13]. Robotic systems can be combined with BCI to provide movement-related feedback. BCI acquires and decodes cerebral activity signals related to movement intentions, converts them into driving commands for external devices, and establishes a closed-loop feedback associated with the stroke patient’s movement intentions, thereby driving the robot to move the affected limbs and promoting post-stroke motor function recovery [14,15].

Buch et al. (2008) conducted early exploratory research on BCI in the field of stroke, and confirmed that chronic stroke patients could control hand orthoses via MEG-BCI to perform grasping movements, indicating that severely paralyzed patients still have the potential to use brain signals to drive external devices [16]. Then a double-blind sham-controlled study confirmed that combining BCI training with physical therapy further enhanced the rehabilitation outcomes for patients with severe chronic stroke, providing direct evidence for the clinical application of BCI in stroke rehabilitation [17]. Later, a multicenter RCT by Frolov et al. demonstrated that motor imagery-based BCI combined with hand exoskeleton training improved post-stroke upper limb motor functions, and the brain signal decoding accuracy was positively correlated with the degree of functional recovery [15]. An RCT from Cheng et al. (2020) further showed that the combined application of motor imagery-based BCI and soft robotic gloves had the potential to promote upper limb functions in chronic stroke patients [12]. Overall, these findings have advanced the application of BCI in post-stroke motor rehabilitation. The intervention format has evolved from early-stage simple motor intention recognition to a closed-loop training mode integrated with orthoses and robotic systems. However, its clinical efficacy still lacks consistent conclusions, particularly in the use of BCI-controlled hand robotics technology in stroke rehabilitation. Studies suggest that BCI-controlled hand robot training outperforms non-BCI-controlled hand robot training in improving upper limb functions in stroke patients [18]. However, Ang’s et al. three-arm RCT showed no significant difference between BCI-controlled and non-BCI-controlled hand robot training in improving upper limb motor function in stroke patients. Nevertheless, compared with conventional rehabilitation therapy, BCI-controlled hand robot training showed more significant improvements at certain time points [19].

Multiple systematic reviews have investigated BCI-based stroke rehabilitation. Early studies mainly focused on the technical feasibility, neural mechanisms, and developmental status of robot-assisted rehabilitation systems, while quantitative evaluations of clinical efficacy remained relatively limited [20,21,22,23]. Among them, Baniqued et al. [20] and Liu et al. [23] summarized the technological development of the field from the perspectives of the technical characteristics of BCI-controlled hand robotic systems, as well as the control strategies and human–computer interaction of hand rehabilitation robots. Robinson et al. [21] and Mane et al. [22], respectively, explored the clinical value of BCI in stroke rehabilitation from the perspectives of clinical efficacy and neural mechanisms. Subsequent meta-analyses suggested that BCI-assisted rehabilitation may improve upper limb motor function after stroke; however, several limitations remain in the existing evidence. For example, Bai et al. [24] included BCI interventions combined with different external devices and performed subgroup analyses according to device type, making it difficult to specifically determine the independent rehabilitation effects of BCI-controlled hand robots. Although Qu et al. [25] focused on BCI-robot systems, the included studies were not rigorously selected based on dose-matching principles.

In contrast, the present study adopted dose-matched inclusion criteria, with total training time between the experimental and control groups treated as a key controlled factor. The effects of post-stroke rehabilitation are influenced not only by the type of intervention but also by the training dose; longer training duration itself may contribute to functional recovery. Such mismatches in total training time may interfere with the accurate estimation of the BCI effect: when the total training time in the BCI group exceeds that of the control group, the therapeutic effect of BCI may be overestimated; conversely, if actual effective training time is reduced in the BCI group due to device preparation or greater task difficulty, its effect may be underestimated. Therefore, in studies without dose-matched designs, the observed between-group effects may simultaneously reflect the specific effects of BCI-driven training and the non-specific effects of additional training dose, thereby reducing the accuracy of meta-analytic conclusions when evaluating the specific intervention of BCI-controlled hand robots. Based on this rationale, the present study adopted total training time-matched inclusion criteria to separate these two effects. In addition, this study focused exclusively on hand robots, thereby reducing the clinical heterogeneity caused by different external devices and training sites and improving the comparability of intervention effects. Therefore, this systematic review and meta-analysis aimed to systematically evaluate the effectiveness of BCI-controlled hand robot training on post-stroke upper limb motor function, particularly hand function recovery, based on dose-matched randomized controlled trials, and to provide more targeted and reliable evidence for its clinical application.

## 2. Methods

This study was registered in PROSPERO (CRD420261348760; registration date: 24 March 2026). The literature search was completed on 13 March 2026, and PROSPERO registration was completed before formal data extraction, risk-of-bias assessment, and data analysis were initiated. This review was reported in accordance with the PRISMA 2020 statement, and the completed checklist is provided in Supplementary Table S1.

### 2.1. Search Strategy

We comprehensively searched English databases including Web of Science, Embase, PubMed, and Cochrane Library, and Chinese databases including CNKI, SinoMed, Wanfang Data, and VIP Database. Additionally, we screened the reference lists of all included studies and relevant reviews as supplementary search. The search covered the period from inception to 13 March 2026, and the key terms included “brain-computer interface,” “robot”, and “stroke” as well as relevant expanded terms. The specific search strategy is listed in Supplementary Table S2.

### 2.2. Eligibility Criteria

Two authors (S.H. and F.J.W.) independently screened all retrieved studies. After duplicate removal, irrelevant studies were first excluded based on titles and abstracts. Full texts of potentially eligible studies were then assessed according to the predefined inclusion and exclusion criteria to determine final eligibility. Any disagreements were resolved through discussion with a third author (X.X.G.).

#### 2.2.1. Inclusion Criteria

(1) RCT; (2) full-text peer-reviewed journal articles published in English or Chinese; (3) patients with clear diagnosis of stroke confirmed by imaging or clinical examination; (4) presence of upper limb motor dysfunction; (5) basically intact cognitive function, able to understand and execute training instructions; (6) the test group receiving hand robot training controlled by BCI; (7) the control group receiving hand robot training under non-BCI conditions or routine rehabilitation therapy (including exercise therapy, occupational therapy, hand function-specific training, upper limb activity training); (8) the test and control groups dose-matched in terms of total training time; (9) the included studies reporting at least one upper limb functional outcome measure: Fugl-Meyer Assessment of Upper Extremity (FMA-UE), Action Research Arm Test (ARAT), FMA-UE proximal score (shoulder/elbow/forearm, 0–42 points), FMA-UE distal score (wrist/hand, 0–24 points), or MAS (finger flexors) score.

#### 2.2.2. Exclusion Criteria

(1) Having other neurological diseases (e.g., Parkinson’s disease, multiple sclerosis, spinal cord injury); (2) thesis or conference abstract; (3) duplicate studies; (4) studies with unavailable full texts; (5) studies with incomplete raw data and no response from authors.

### 2.3. Data Extraction

Two authors (S.H. and F.J.W.) independently extracted data from the included studies, including (1) characteristics of the study subjects (author, publication year, patient age, trial sample size, mean disease duration, stroke stage, severity of upper limb dysfunction); (2) intervention methods (type of intervention, training dose, type of outcome measure); (3) indicators reflecting study quality; (4) outcome measure results. After data extraction, two authors (S.H. and F.J.W.) cross-checked the information. Any discrepancy was resolved through discussion with a third author (X.X.G.) to reach consensus.

### 2.4. Quality Assessment and Certainty of Evidence

Two authors (S.H. and F.J.W.) assessed the quality of the included studies using the Cochrane Risk of Bias (ROB) tool, including random sequence generation, allocation concealment, blinding, incomplete outcome data, selective reporting, and other potential sources of bias. Any disagreements were resolved by discussion with the third author.

In addition, the certainty of evidence for each outcome was assessed using the GRADE (Grading of Recommendations Assessment, Development and Evaluation) approach. This method evaluates the certainty of evidence across five domains: risk of bias, inconsistency, indirectness, imprecision, and publication bias. The certainty of evidence was classified into four levels: high, moderate, low, and very low.

### 2.5. Statistical Analysis

#### 2.5.1. Calculation of Effect Size

Statistical analysis was performed using Review Manager 5.4. The outcome indicators were all continuous variables, and mean difference (MD) was used for pooled analysis. Effect sizes were expressed as 95% confidence interval (CI); differences were considered significant at *p* < 0.05. Continuous variables reported as median and interquartile range were converted to mean and standard deviation using Wan et al.’s method [26].

#### 2.5.2. Heterogeneity Analysis

Where I^2^ values of 25%, 50% and 75% indicate low, moderate, and high heterogeneity, respectively [27]. Considering the potential clinical heterogeneity among the included studies, including differences in stroke stage, robot type, and BCI paradigm, and given the limited statistical power of the I^2^ statistic when the number of included studies is small (*n* = 11), a random-effects model was used by default for all meta-analyses to provide more conservative effect estimates. The I^2^ value was mainly used to describe the degree of heterogeneity rather than serve as a threshold for model selection. To further explore potential sources of clinical heterogeneity and verify the reliability of the results, subgroup analyses and sensitivity analyses using the leave-one-out method were conducted.

#### 2.5.3. Publication Bias

Since fewer than 10 original studies were included for each outcome measure, the statistical power of funnel plots to detect publication bias was limited, and thus this test was not performed here. Instead, we examined the reference lists of all included studies and relevant reviews to identify potentially omitted literature. This approach follows the recommendation from the Cochrane Handbook to avoid methods with limited statistical power for assessing publication bias when the number of included studies is small.

#### 2.5.4. Subgroup Analysis

Subgroup analyses of the primary outcome measures FMA-UE and ARAT were conducted on basis of stroke phase (subacute, chronic), robot type (exoskeleton, soft robotic glove, end-effector), BCI paradigm type (MI-BCI, MA-BCI, SSVEP-BCI), upper limb impairment severity (moderate to severe, severe), and follow-up duration (6, 12, 18 weeks).

### 2.6. Outcome Indicators

This study focused on the recovery of hand function, specifically targeting the post-stroke rehabilitation of upper limb motor impairments, and thus primarily used the included outcome measures to evaluate overall upper limb motor function, manual dexterity, and related functional improvements in patients.

#### 2.6.1. Primary Outcome Indicators

Fugl-Meyer Assessment for Upper Extremity (FMA-UE): It is primarily used to evaluate the overall motor impairment and recovery of the upper extremity in stroke patients, and has a total score from 0 to 66, where higher scores indicate better overall motor function of the upper extremity. Recent psychometric systematic reviews indicate that the FMA-UE is one of the most commonly used and highly recommended tools for assessing upper extremity motor function after stroke [28].

Action Research Arm Test (ARAT): It is mainly used to assess the upper limb and hand manipulation abilities of stroke patients in tasks such as grasping, gripping, pinching, and gross movements. This scale consists of 19 items with a total score of 0–57, where higher scores indicate better ability. ARAT is an effective and reliable tool for evaluating upper limb and hand functions in stroke patients [29].

#### 2.6.2. Secondary Outcome Indicators

FMA-UE proximal score: It is primarily used to assess the motor function of proximal upper limb joints (e.g., shoulder and elbow) in stroke patients, and has a total score from 0 to 42. In stroke rehabilitation research, the FMA-UE is often divided into proximal and distal subscales to reflect the recovery of different upper limb segments separately [28].

FMA-UE distal score: It is mainly used to evaluate the motor function of distal segments (e.g., wrist and hand) in stroke patients, and effectively reflects the recovery of hand-related movements, with a total score from 0 to 24 [28].

MAS (finger flexor) score: It is used to assess the degree of spasticity or increased muscle tone in the finger flexors of stroke patients, and has a total of 6 rating levels (0, 1, 1+, 2, 3, 4). A higher score indicates more severe increased muscle tone [30].

## 3. Results

### 3.1. Research Results

**Figure 1:**
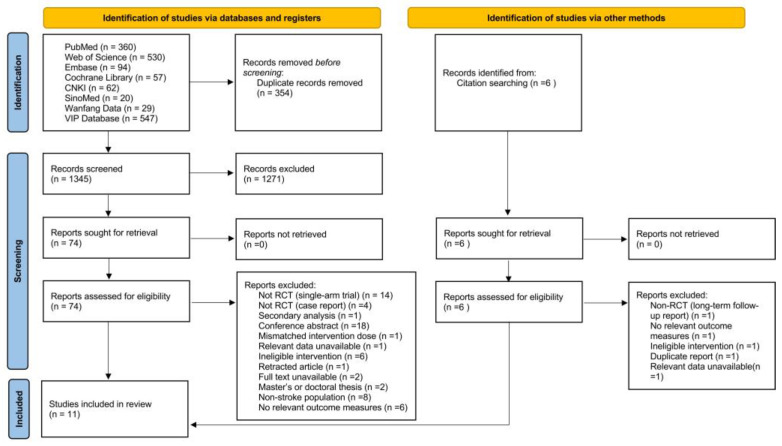
PRISMA flow diagram of literature screening.

Totally 1705 articles were retrieved, including 1041 articles from English databases, 658 articles from Chinese databases, and 6 articles from other sources (reference tracing). After removing 354 duplicate articles using EndNote 21, 1345 articles remained for title and abstract screening. After reviewing the titles and abstracts, 1271 irrelevant articles were excluded, leaving 74 articles for full-text screening. Together with 6 additional articles identified through reference tracing, a total of 80 articles underwent full-text screening. After full-text screening, 69 studies that did not meet the inclusion criteria were excluded (specific exclusion reasons and literature list are detailed in Supplementary Table S3), resulting in the inclusion of 11 RCTs. The literature screening process is illustrated in Figure 1.

### 3.2. Characteristics of Included Studies

Totally 11 studies involving 380 stroke patients were included, with 202 cases in the test group and 178 cases in the control group. The age of the included stroke patients mainly ranged from 51 to 65 years. The stroke stages covered the subacute and chronic phases, with 7 studies including subacute patients, 6 studies including chronic patients, 3 studies including both stages, and 1 study not reporting the stroke stage. Detailed characteristics of the participants in the included studies are shown in Table 1.

As for intervention characteristics, the majority of the included studies utilized MI-BCI, only one study employed SSVEP-BCI and another study adopted MA-BCI. The test group received BCI-controlled hand robot training, while the control group underwent sham BCI-controlled hand robot training or conventional rehabilitation therapy. The studies generally adopted a dose-matched design, and the single training session duration ranged from 30 to 120 min, with a training frequency mostly of 3–5 times per week and an intervention duration of 2–6 weeks. Outcome assessment time points typically included baseline and post-intervention, and some studies further reported follow-up results at 6, 12, or 18 weeks. As for outcome measures, all 11 studies reported FMA-UE, and 6, 3, 4, and 2 studies reported ARAT, FMA-UE proximal, FMA-UE distal, and MAS-finger respectively. Detailed intervention characteristics of the included studies are presented in Table 2.

### 3.3. Risk of Bias Assessment in Included Studies

**Figure 2:**
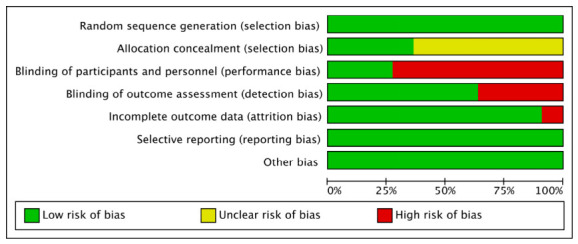
Proportional distribution of bias risk types.

**Figure 3:**
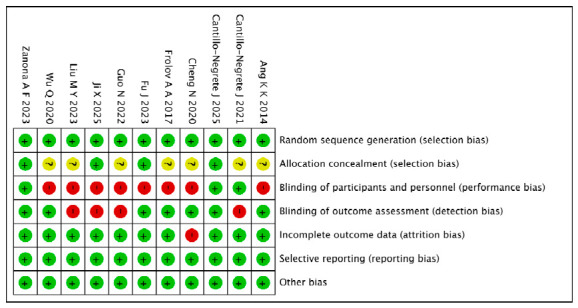
Summary of bias risk in included studies. Green, yellow and red circles indicate low, questionable, and high risk of bias respectively.

Among the 11 included studies, all 11 studies (100%) had a low risk of bias in random sequence generation. Allocation concealment was reported in 4 studies (36.36%), but not in the remaining 7 studies (63.64%). Due to the evident operational nature of BCI-robot training, 8 studies (72.73%) did not implement blinding for investigators and participants, and only 3 studies (27.27%) applied blinding to both participants and investigators. In terms of outcome assessor blinding, 7 studies (63.64%) utilized blinding for outcome assessors, whereas 4 studies (36.36%) were rated as high risk. In terms of the completeness of outcome data, 10 studies (90.91%) had complete outcome data, while 1 study (9.09%) did not specify the reasons for loss to follow-up. All 11 studies (100%) had no selective reporting of results. In terms of other biases, no significant risks were observed in any of the studies. The results of the bias risk assessment are shown in Figure 2 and Figure 3.

### 3.4. Results of Primary Outcome Indicators

#### 3.4.1. FMA-UE

BCI-controlled hand robot training significantly improved the FMA-UE scores of stroke patients compared to non-BCI-controlled hand robot training (MD = 4.87, 95% CI 1.04–8.69, *p* = 0.01), without significant inter-study heterogeneity (I^2^ = 0%, *p* = 0.54) (Supplementary Figure S1). BCI-controlled hand robot training also significantly enhanced the FMA-UE scores of stroke patients compared to conventional rehabilitation (MD = 6.55, 95% CI 3.49 to 9.61, *p* < 0.0001), with low inter-study heterogeneity (I^2^ = 1%, *p* = 0.41) (Supplementary Figure S2).

#### 3.4.2. ARAT

Compared to non-BCI-controlled hand robot training, BCI-controlled hand robot training showed no significant difference in improving ARAT scores in stroke patients (MD = 1.87, 95% CI −4.01 to 7.75, *p* = 0.53) (Supplementary Figure S3), with low heterogeneity (I^2^ = 0%, *p* = 0.4). However, due to the limited number of included studies (*n* = 4 studies), these results should be interpreted with caution.

Compared with conventional rehabilitation, only two studies reported ARAT scores [31,34]. After pooling these studies, extremely high heterogeneity was observed (I^2^ = 84%, *p* = 0.01) (Supplementary Figure S4); therefore, a descriptive analysis was performed. One study showed that both BCI therapy (8.4 ± 10) and conventional therapy (8.7 ± 11) significantly improved ARAT scores from baseline levels (4.3 ± 6) (*p* < 0.017 after Bonferroni correction), but there was no significant difference between the two treatment regimens (*p* > 0.017 after Bonferroni correction) [31]. Another study showed that ARAT scores significantly improved from baseline in both the BCI group (*n* = 14) and the conventional rehabilitation group (*n* = 11) (BCI group: *p* = 0.001; conventional rehabilitation group: *p* = 0.011), and that the improvement was significantly greater in the BCI group than in the conventional rehabilitation group (between-group comparison: *p* = 0.045). However, this study had a small sample size, and the findings should therefore be interpreted with caution [34].

### 3.5. Results of Secondary Outcome Indicators

#### 3.5.1. FMA-UE Proximal Score

The meta-analysis results indicate high consistency among the included studies (I^2^ = 0%, *p* = 0.98). BCI-controlled hand robot training was significantly superior over non-BCI-controlled hand robot training in improving FMA-UE proximal scores in stroke patients (MD = 4.44, 95% CI 0.15 to 8.74, *p* = 0.04) (Supplementary Figure S5). Similarly, BCI training also demonstrated significant advantages compared to conventional rehabilitation (MD = 7.92, 95% CI 1.92 to 13.91, *p* = 0.01), with high consistency among studies (I^2^ = 0%, *p* = 0.84) (Supplementary Figure S6).

#### 3.5.2. FMA-UE Distal Score

In improving the FMA-UE distal score, there was no significant difference between BCI-controlled and non-BCI-controlled hand robot training (MD = 0.17, 95% CI −0.71 to 1.04, *p* = 0.71), with no significant inter-study heterogeneity (I^2^ = 0%, *p* = 0.56) (Supplementary Figure S7). However, compared to conventional rehabilitation, BCI-controlled hand robot training showed better efficacy (MD = 5.06, 95% CI 1.05 to 9.07, *p* = 0.01), with low statistical heterogeneity (I^2^ = 0%, *p* = 0.97) (Supplementary Figure S8).

#### 3.5.3. MAS (Finger Flexor)

Training with BCI-controlled hand robots significantly reduced the MAS (finger flexor) score compared to training with non-BCI-controlled hand robots (MD = −0.44, 95% CI −0.68 to −0.21, *p* = 0.0002), with low statistical heterogeneity observed between studies (I^2^ = 0%, *p* = 0.33) (Supplementary Figure S9).

### 3.6. Subgroup Analysis Results

#### 3.6.1. Stroke Stage

**Figure 4:**
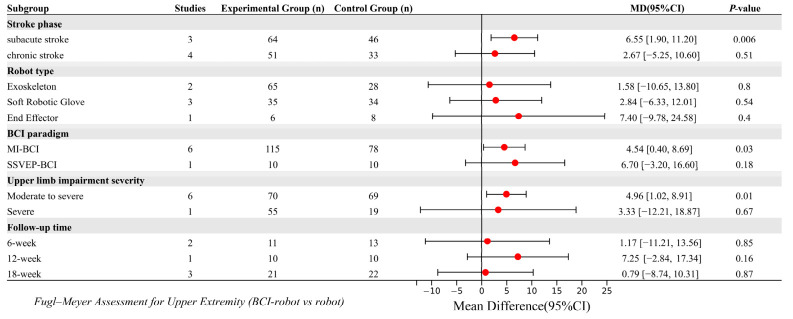
Forest plot of subgroup analysis of FMA-UE: BCI-robot vs. robot. Red dots indicate the point estimates of the mean difference (MD), and horizontal lines represent the corresponding 95% confidence intervals (CIs).

For the FMA-UE score, compared with non-BCI-controlled hand robot training, BCI-controlled hand robot training showed significant improvement only in subacute stroke patients (MD = 6.55, 95% CI 1.90 to 11.20, *p* = 0.006), with low statistical heterogeneity (I^2^ = 0%, *p* = 0.96). In contrast, no significant difference was found between the two groups in chronic stroke patients (MD = 2.67, 95% CI −5.25 to 10.60, *p* = 0.51), with low heterogeneity (I^2^ = 19%, *p* = 0.29) (Figure 4 and Figure S10).

**Figure 5:**
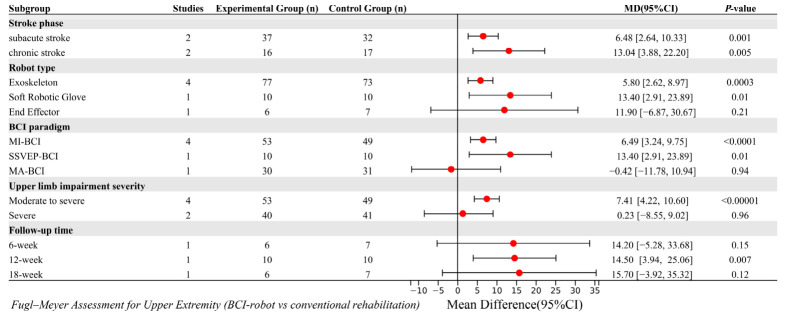
Forest plot of subgroup analysis of FMA-UE: BCI-robot vs. conventional rehabilitation. Red dots indicate the point estimates of the mean difference (MD), and horizontal lines represent the corresponding 95% confidence intervals (CIs).

Compared with conventional rehabilitation, BCI-controlled hand robotic training significantly improved FMA-UE scores in both subacute stroke patients (MD = 6.48, 95% CI 2.64 to 10.33, *p* = 0.001; I^2^ = 7%, *p* = 0.30) and chronic stroke patients (MD = 13.04, 95% CI 3.88 to 22.20, *p* = 0.005; I^2^ = 0%, *p* = 0.89) (Figure 5 and Figure S11).

**Figure 6:**
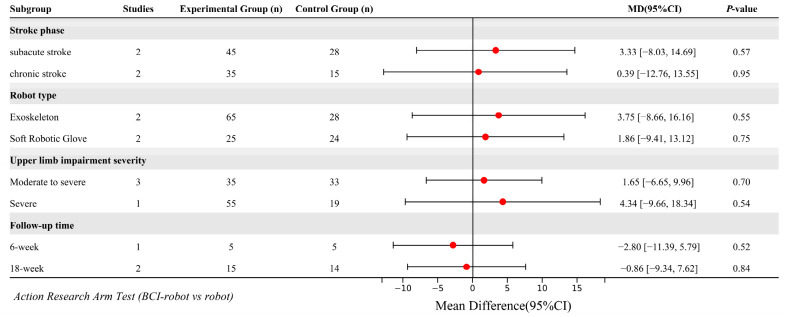
Forest plot of subgroup analysis of ARAT: BCI-robot vs. robot. Red dots indicate the point estimates of the mean difference (MD), and horizontal lines represent the corresponding 95% confidence intervals (CIs).

For the ARAT score, BCI-controlled hand robot training did not demonstrate significant improvement compared to non-BCI-controlled hand robot training in either subacute or chronic patients. The results were subacute phase (MD = 3.33, 95% CI −8.03 to 14.69, *p* = 0.57, I^2^ = 39%, *p* = 0.20), and chronic phase (MD = 0.39, 95% CI −12.76 to 13.55, *p* = 0.95, I^2^ = 35%, *p* = 0.22). Heterogeneity ranged from low to moderate across the subacute and chronic subgroups. (Figure 6 and Figure S12).

#### 3.6.2. Type of Robot

For the FMA-UE score, no significant differences were observed between BCI-controlled hand robot training and non-BCI-controlled hand robot training across different robot types: exoskeleton robots (MD = 1.58, 95% CI −10.65 to 13.80, *p* = 0.80, I^2^ = 0%, *p* = 0.72), soft robotic gloves (MD = 2.84, 95% CI −6.33 to 12.01, *p* = 0.54, I^2^ = 50%, *p* = 0.14), and end-effectors (*n* = 1 study, MD = 7.40, 95% CI −9.78 to 24.58, *p* = 0.40). Notably, low statistical heterogeneity was observed in exoskeleton robots, while moderate heterogeneity was present in soft robotic gloves (Figure 4 and Figure S13).

Compared to conventional rehabilitation, BCI-controlled hand robot training demonstrated significant improvements in the soft robotic glove group (*n* = 1 study, MD = 13.40, 95% CI 2.91 to 23.89, *p* = 0.01) and the exoskeleton robot group (MD = 5.80, 95% CI 2.62 to 8.97, *p* = 0.0003, I^2^ = 0%, *p* = 0.41). However, no clear statistical difference was observed in the end-effector group (*n* = 1 study, MD = 11.90, 95% CI −6.87 to 30.67, *p* = 0.21). Due to the limited number of studies, the effect may be unstable, warranting further investigation (Figure 5 and Figure S14).

For the ARAT score, BCI-controlled hand robot training did not demonstrate significant improvement compared to non-BCI-controlled hand robot training in either the exoskeleton robot group (MD = 3.75, 95% CI −8.66 to 16.16, *p* = 0.55; I^2^ = 0%, *p* = 0.86) or the soft robotic glove group (MD = 1.86, 95% CI −9.41 to 13.12, *p* = 0.75; I^2^ = 64%, *p* = 0.09). Heterogeneity was low in the exoskeleton robot group and moderate in the soft robotic glove group (Figure 6 and Figure S15).

#### 3.6.3. Types of BCI Paradigms

For the FMA-UE score, MI-BCI-controlled hand robot training significantly improved FMA-UE scores in stroke patients compared to non-BCI-controlled hand robot training (MD = 4.54, 95% CI 0.40 to 8.69, *p* = 0.03), with low statistical heterogeneity observed (I^2^ = 0%, *p* = 0.44). Although the SSVEP-BCI subgroup showed a trend of improvement, but not significant (*n* = 1 study, MD = 6.70, 95% CI −3.20 to 16.60, *p* = 0.18) (Figure 4 and Figure S16).

Compared to conventional rehabilitation, MI-BCI-controlled hand robot training significantly improved the FMA-UE scores in stroke patients (MD = 6.49, 95% CI 3.24 to 9.75, *p* < 0.0001), with low statistical heterogeneity observed (I^2^ = 0%, *p* = 0.58). SSVEP-BCI also demonstrated significant improvement (*n* = 1 study, MD = 13.40, 95% CI 2.91 to 23.89, *p* = 0.01), but no significant difference was found with MA-BCI (*n* = 1 study, MD = −0.42, 95% CI −11.78 to 10.94, *p* = 0.94) (Figure 5 and Figure S17).

#### 3.6.4. Degree of Upper Limb Impairment

For FMA-UE scores, compared with non-BCI-controlled hand robot training, BCI-controlled hand robot training significantly improved FMA-UE scores in patients with moderate-to-severe upper limb impairment (MD = 4.96, 95% CI 1.02 to 8.91, *p* = 0.01), with low statistical heterogeneity observed (I^2^ = 0%, *p* = 0.42). However, in patients with severe upper limb impairment, no significant difference was found between the two groups (*n* = 1 study, MD = 3.33, 95% CI −12.21 to 18.87, *p* = 0.67) (Figure 4 and Figure S18).

Compared to conventional rehabilitation, BCI-controlled hand robot training significantly improved FMA-UE scores in patients with moderate-to-severe upper limb impairment (MD = 7.41, 95% CI 4.22 to 10.60, *p* < 0.00001) without significant heterogeneity (I^2^ = 0%, *p* = 0.43). No significant difference was observed in patients with severe upper limb impairment (MD = 0.23, 95% CI −8.55 to 9.02, *p* = 0.96, I^2^ = 0%, *p* = 0.86) (Figure 5 and Figure S19).

For the ARAT score, compared with non-BCI-controlled hand robot training, BCI-controlled hand robot training did not demonstrate significant improvement effects in patients with moderate-to-severe upper limb impairment (MD = 1.65, 95% CI −6.65 to 9.96, *p* = 0.70, I^2^ = 28%, *p* = 0.25) or in those with severe upper limb impairment (*n* = 1 study, MD = 4.34, 95% CI −9.66 to 18.34, *p* = 0.54). Notably, the moderate-to-severe upper limb impairment subgroup exhibited low-to-moderate heterogeneity. (Figure 6 and Figure S20).

#### 3.6.5. Follow-Up Time

For the long-term follow-up results of FMA-UE scores, compared with non-BCI-controlled hand robot training, BCI-controlled hand robot training did not demonstrate significant improvement at 6 weeks (MD = 1.17, 95% CI −11.21 to 13.56, *p* = 0.85, I^2^ = 31%, *p* = 0.23), 12 weeks (*n* = 1 study, MD = 7.25, 95% CI −2.84 to 17.34, *p* = 0.16), or 18 weeks (MD = 0.79, 95% CI −8.74 to 10.31, *p* = 0.87, I^2^ = 0%, *p* = 0.55). The overall pooled results also showed no significant difference (MD = 2.74, 95% CI −2.94 to 8.43, *p* = 0.34), with low statistical heterogeneity between studies (I^2^ = 0%, *p* = 0.58) (Figure 4 and Figure S21).

Compared to conventional rehabilitation, BCI-controlled hand robot training significantly improved FMA-UE scores at the 12-week follow-up (*n* = 1 study, MD = 14.50, 95% CI 3.94 to 25.06, *p* = 0.007). No significant differences were observed at the 6-week (*n* = 1 study, MD = 14.20, 95% CI −5.28 to 33.68, *p* = 0.15) or 18-week follow-up (*n* = 1 study, MD = 15.70, 95% CI −3.92 to 35.32, *p* = 0.12). The overall pooled results demonstrated that BCI-controlled hand robot training significantly enhanced FMA-UE scores (MD = 14.66, 95% CI 6.27 to 23.05, *p* = 0.0006), with low statistical heterogeneity observed across studies (I^2^ = 0%, *p* = 0.99) (Figure 5 and Figure S22).

For the long-term follow-up results of ARAT scores, compared to non-BCI-controlled hand robot training, BCI-controlled hand robot training did not demonstrate significant improvement at the 6-week follow-up (*n* = 1 study, MD = −2.80, 95% CI −11.39 to 5.79, *p* = 0.52). Similarly, no significant difference was observed at the 18-week follow-up (MD = −0.86, 95% CI −9.34 to 7.62, *p* = 0.84), with low statistical heterogeneity (I^2^ = 0%, *p* = 0.82). The overall pooled results showed no significant difference (MD = −1.82, 95% CI −7.85 to 4.22, *p* = 0.55), with low statistical heterogeneity observed among studies (I^2^ = 0%, *p* = 0.93) (Figure 6 and Figure S23).

### 3.7. GRADE Assessment of Certainty of Evidence

GRADE assessment showed that the certainty of evidence for all outcomes ranged from low to very low. Specifically, the certainty of evidence for FMA-UE, FMA-UE proximal, and FMA-UE distal was rated as low, whereas ARAT and MAS-finger were rated as very low (Supplementary Table S4).

## 4. Discussion

We systematically evaluated the effects of BCI-controlled hand robot training on upper limb rehabilitation focusing on hand function after stroke. Compared with previous systematic reviews and meta-analyses, the present study also supports that BCI-based interventions can improve post-stroke upper limb motor function. However, unlike most previous studies, Baniqued et al. [20] and Liu et al. [23] mainly focused on technical feasibility and control strategies, while Robinson et al. [21] and Mane et al. [22] more broadly addressed the assistive and holistic rehabilitation applications of BCI. In contrast, Bai et al. [24] and Qu et al. [25] included studies with greater heterogeneity in external device types and intervention protocols, and did not adopt dose-matched designs. The present study focused on BCI-controlled hand robot training and adopted dose matching as an inclusion criterion, which reduced, to some extent, the confounding effects related to clinical heterogeneity and differences in training dose, thereby helping to evaluate the specific rehabilitation effects of BCI-controlled hand robot training.

BCI-controlled hand robot training showed potential advantages in overall upper limb motor function (FMA-UE) and proximal function (FMA-UE proximal) compared to robot-only training or conventional rehabilitation. Specifically, compared with robot-only training (MD = 4.87, 95% CI 1.04 to 8.69, *p* = 0.01) or conventional rehabilitation (MD = 6.55, 95% CI 3.49 to 9.61, *p* < 0.0001), FMA-UE scores were significantly improved. The corresponding point estimates exceeded the previously reported minimal clinically important difference (MCID) threshold (≥4 points) [38], suggesting potential clinical relevance. However, the lower bounds of both confidence intervals were below this threshold, and therefore the clinical significance should still be interpreted cautiously. However, compared with robot-only training, no significant additional benefits were observed in distal upper limb function (FMA-UE distal) or manual dexterity (ARAT).

Previous meta-analyses on BCI combined with robot intervention show certain inconsistency, which may be related to differences in intervention forms and study designs. The meta-analysis by Qu et al. suggests no significant difference between the BCI-robot group and the robot-only group in improving FMA-UE scores (*p* > 0.05) [25]. This study included robot intervention sites with a lack of consistency and did not use dose matching as an inclusion criterion, potentially underestimating the true effect of BCI intervention. The meta-analysis by Li et al. found that BCI combined with rehabilitation training (e.g., robots, functional electrical stimulation, virtual reality) significantly improved FMA-UE scores in stroke patients. However, its subgroup analysis showed that the combined intervention of BCI and robot did not significantly improve patients in the subacute phase (*p* = 0.34), with extremely high heterogeneity (I^2^ = 89%), while it was significantly effective for chronic-phase patients (*p* = 0.03) [39]. This finding differs from the present study: under dose-matched conditions, we found that BCI-controlled hand robot training appeared to be more beneficial than robot training alone for patients in the subacute phase (*p* = 0.006). Specifically, the point estimate for FMA-UE improvement (MD = 6.55) exceeded the reported MCID threshold (≥4 points) [38], whereas the lower bound of the 95% confidence interval (1.90) did not reach this threshold, suggesting possible interindividual variability in clinical benefit. Therefore, the clinical significance should still be interpreted cautiously. In contrast, no significant difference was observed in chronic-phase patients (*p* = 0.51). This null finding suggests that for chronic stroke patients, adding BCI control to hand robot training may not confer additional benefits over robot training alone, which is an important consideration for clinical decision-making in this population. Evidence from the Critical Period After Stroke Study (CPASS) clinical trial showed that upper limb motor rehabilitation initiated within 60–90 days after stroke led to the greatest improvement, whereas training initiated at 6 months or later was associated with markedly reduced additional benefits [40]. This phenomenon suggests that upper limb motor recovery after stroke may involve a time-dependent plasticity window [40,41]. From a neurophysiological perspective, activity-dependent neuroplasticity is more active in patients in the subacute phase, which is more favorable for Hebbian-type plasticity. In addition, the remaining sensorimotor pathways may be more readily reactivated and strengthened through training [41,42]. Therefore, BCI-controlled robotic training, by coupling movement intention with sensory feedback, may more easily promote the reconstruction of the sensorimotor loop in the subacute phase [42]. In contrast, in the chronic phase, this plasticity window may have partially closed [40], leading to reduced responsiveness of the intention-driven closed-loop feedback mechanism and thereby limiting the recovery effect of BCI training in the chronic phase. However, given the limited number of included chronic-phase studies, this result should be interpreted with caution. Moreover, compared to conventional rehabilitation, significant improvement was observed in both subacute-phase patients (MD = 6.48, 95% CI 2.64 to 10.33, *p* = 0.001) and chronic-phase patients (MD = 13.04, 95% CI 3.88 to 22.20, *p* = 0.005). Although the point estimates in both subgroups exceeded their corresponding MCID thresholds (subacute phase ≥ 4 points [38]; chronic phase ≥ 5.25 points [43]), the lower bounds of the confidence intervals did not reach the MCID thresholds, suggesting that the clinical significance should still be interpreted cautiously. The reason for this discrepancy may be that Li et al. combined robot training with conventional rehabilitation as the control group, which led to mixed interventions and high heterogeneity and thereby affected the results. Zhou’s et al. network meta-analysis demonstrated that BCI-based rehabilitation robots ranked first in both overall effect (SUCRA = 99.9%) and short-term effect (≤4 weeks, SUCRA = 99.4%) in improving upper limb function post-stroke (FMA-UE scores), significantly outperforming conventional therapy and robot training. However, in terms of long-term effects (>4 weeks), although the BCI robot still ranked first in SUCRA (85.1%), the difference compared to conventional therapy did not reach statistical significance [44]. This result indicates that the advantage of BCI robots primarily manifests in short-term interventions, and the sustainability of its benefits over time remains to be verified.

We found that previous studies often combined different feedback modalities (e.g., BCI combined with functional electrical stimulation, virtual reality, or robots for analysis), so their results primarily reflect the comprehensive effects of BCI overall intervention. The lack of intervention homogeneity makes it impossible to more specifically reflect the actual gain effect when BCI drives a specific type of external devices [45]. Therefore, this study focuses on BCI-controlled hand robot training and adopts a dose-matching design, which improves intervention homogeneity to some extent and enhances the internal consistency and explanatory power of the research findings. However, combined BCI interventions are still in the exploratory phase, as there are only a limited number of high-quality, dose-matched RCTs [20]. Therefore, the dose-matching criterion in this study only ensured equal total training time between the experimental and control groups, thereby controlling the overall treatment load rather than matching the specific composition of training content. Compared with previous meta-analyses without dose matching, the present study minimized the influence of inconsistent training dosage on effect estimation by strictly controlling total training time, thereby allowing a more accurate evaluation of the rehabilitation effect of BCI-controlled hand robot training. However, it should also be acknowledged that, although total training time was matched between groups, active BCI training in the experimental group accounted for only part of the total session duration, and differences in the quality and specificity of training content may still have existed between groups. In particular, if the supplementary training tasks in the control group were themselves beneficial for upper limb recovery, this may have reduced the between-group difference and thereby underestimated the added value of BCI-controlled hand robot training. This may introduce a specific directional bias under the current total-time dose-matching design.

In addition, although we attempted to enhance comparability by restricting the intervention site to the hand, standardizing the external device type as robotic, and adopting dose-matched non-invasive BCI designs, this study inevitably included studies involving different types of robots, BCI paradigms, and various stages of stroke. Although most outcomes showed low statistical heterogeneity (I^2^ = 0%), this may be related to the small number of included studies, as the I^2^ statistic may have limited power to detect true between-study heterogeneity in meta-analyses with small sample sizes. Therefore, subgroup analyses were conducted to exclude the potential influence of clinical heterogeneity on effect sizes and further clarify the applicable conditions for BCI-controlled hand robot training.

We found that BCI-controlled hand robots were more effective in improving proximal arm function than distal function. The FMA-UE total score and proximal subitems primarily reflect improvements in relatively gross movements, such as flexion-extension, lifting, and synergistic control, whereas distal function and ARAT rely more on grasping, pinching, independent finger control, and sensorimotor integration [46]. These functions typically recover more slowly and require more specific training regimens [47]. Schambra et al. note that the recovery timelines for proximal (e.g., shoulder, elbow) and distal (e.g., fingers) upper limb functions after stroke are not synchronized, as their underlying recovery mechanisms differ [48]. From a neural perspective, distal hand functions, particularly isolated finger movements and fine motor skills, rely more heavily on the integrity of the corticospinal tract. In contrast, proximal shoulder and elbow movements can additionally recruit the reticulospinal pathway and broader synergistic control beyond the corticospinal tract [49,50]. Consequently, when the corticospinal tract is damaged after stroke, proximal motor functions are often preserved or recovered more readily than distal fine movements, making post-stroke recovery of finger and wrist-related functions particularly challenging. However, the number of studies reporting distal upper limb function (FMA-UE distal, ARAT) in this review is limited, and further exploration is needed to determine the effects of BCI-controlled hand robots on post-stroke distal fine motor function.

We found that the improvement in patients with severe upper limb impairment after stroke was not satisfactory and none reached significance, but the limited number of included studies calls for cautious interpretation. The fundamental reason for the difficulty in recovery of severe upper limb impairment after stroke lies in the damage to extensive sensorimotor brain regions, which severely weakens the structural basis on which neuroplasticity depends, limiting rehabilitation outcomes [51]. The existing conventional rehabilitation methods have minimal effects on improving severe upper limb impairment after stroke, and BCI is a promising approach for treating severe upper limb impairment [52]. The potential advantages of BCI combined training may stem not only from mechanical assistance, but also from movement intention-driven and closed-loop feedback mechanisms [53]. The RCT by Ramos-Murguialday et al. demonstrates that BCI training can induce clinically significant improvement in upper limb motor function for patients with severe chronic stroke who lack active finger extension ability (cFMA improvement of 3.41 points, *p* = 0.018) [17]. The mechanism may lie in the fact that BCI instantly associates the patient’s movement intention with limb movement feedback by closing the sensorimotor loop, thereby activates Hebbian plasticity and strengthens the sensorimotor loop on the lesion side, the shift of brain activity toward the injured hemisphere, and the injured hemisphere’s control over the movement of the paralyzed upper limb, ultimately facilitating the recovery of motor function [54].

In terms of the types of BCI paradigms, we found that MI-BCI combined with robots yielded better outcomes. MI-BCI primarily works by identifying rhythmic changes in the sensorimotor cortex during motor imagery, which are typically manifested as event-related desynchronization and synchronization of sensorimotor rhythms [55]. By detecting brain activity induced by motor imagery and converting it into commands for external devices or response systems, this feedback loop helps reinforce motor learning and improve classification accuracy. Both the optimal classification accuracy and the average classification accuracy are significantly correlated with upper limb functional improvement, which may be one key reason for the superior efficacy of MI-BCI combined with robotic training [7,15]. In addition, another BCI paradigm included in the present study, namely SSVEP-BCI, also demonstrated certain rehabilitation potential. Compared with conventional rehabilitation, SSVEP-BCI intervention significantly improved FMA-UE scores (MD = 13.40, 95% CI 2.91 to 23.89, *p* = 0.01), and the between-group difference exceeded the reported MCID threshold for post-stroke FMA-UE scores (≥4 points) [38]. SSVEP-BCI is an exogenous visual-attention-dependent brain-computer interface paradigm and is fundamentally different from MI-BCI, which decodes motor imagery-related cortical activity. SSVEP-BCI has several advantages, including a relatively high signal-to-noise ratio and shorter training-time requirements, and may therefore be feasible and clinically valuable for stroke patients with limited motor imagery ability [56]. In the SSVEP study included in the present review, the decoded visual-attention signals were used to trigger movements of a soft robotic glove, thereby linking goal-directed visual attention with repetitive hand training and synchronous visual-proprioceptive feedback [33]. Such feedback may strengthen visuomotor coupling and sensorimotor integration, which are important for the reconstruction of upper limb motor control after stroke, particularly when visual information is used to compensate for proprioceptive deficits [57]. From a neurophysiological perspective, visual feedback is not merely an external cue. Instead, it engages visual–parietal–motor-related networks involved in action selection, motor correction, and motor learning, thereby facilitating functional activation of the affected motor cortex [58]. In addition, combining visual information with physical training may enhance post-stroke motor rehabilitation and is associated with selective enhancement of cortical motor excitability [59]. Collectively, these mechanisms suggest that the “visual attention–robotic movement–sensory feedback” loop established by SSVEP-BCI robotic training may facilitate hand function recovery through enhanced sensorimotor integration. It should be noted that only one SSVEP-related study was included in the present systematic review; therefore, the findings of this subgroup analysis should be considered exploratory and require further validation.

In addition, although the pooled effect showed a statistically significant reduction in MAS-finger scores (MD = −0.44), this average change was below the previously reported MCID range for upper-limb MAS in stroke (0.48–0.76 points) [60]; therefore, whether this finding represents a clinically meaningful improvement in spasticity remains uncertain. Moreover, the certainty of evidence for this outcome was very low, and the conclusion still requires further validation by future studies. In contrast, the meta-analysis by Mortezaei et al. did not find an overall improvement in MAS with BCI training, but the studies included in the analysis exhibited considerable heterogeneity in terms of external device types, intervention frequency, and total duration. This inconsistency suggests that the efficacy of BCI on spasticity is not universally established and may depend on specific external feedback devices [61].

In the long-term follow-up, we observed that the BCI-controlled hand robot significantly outperformed conventional rehabilitation in FMA-UE scores only at the 12-week mark. However, this finding was based on only one included study and should therefore be interpreted with caution. Lau et al. employed a single-group pre-post design, and after 20 sessions of BCI-guided hand robot training in 14 chronic stroke patients, FMA-UE and ARAT scores are still significantly improved at the 6-month follow-up. Meanwhile, resting-state fMRI revealed enhanced neuronal activity in the sensorimotor and frontoparietal regions, a change that persisted for 6 months [62]. However, the maximum follow-up duration in our subgroup was 18 weeks, and no significant improvements were observed in FMA-UE or ARAT scores. The meta-analysis by Chen et al. also indicated that BCI-based training showed no significant advantage in improving FMA-UE scores during short-to-medium-term (≤3 months) or long-term (>3 months) follow-up [63]. The current evidence suggests inconsistent findings regarding the long-term efficacy of BCI-combined training, which remains exploratory. Although the external feedback modality differed from the robotic systems included in the present review, a recent 4.5-year longitudinal study of BCI combined with functional electrical stimulation reported sustained motor improvement in elderly stroke patients and EEG-based neuroplastic changes, further supporting the potential role of closed-loop BCI feedback in long-term neurorehabilitation [64]. Future efforts should focus on further exploration of the long-term effects of BCI-combined training.

## 5. Advantages and Limitations

The strengths of this study are mainly reflected in the following aspects. First, the included studies exhibit high homogeneity, and low statistical heterogeneity exists in most analyzed outcomes (I^2^ = 0%), enhancing the robustness and reliability of the findings. Second, our comprehensive systematic search covered literature from major databases, traced references from relevant reviews, and included both Chinese and English publications, which may help reduce language bias and improve the comprehensiveness of the evidence retrieval.

This study also has some limitations. First, the small number of included studies resulted in insufficient statistical power for subgroup analyses, potentially reducing the reliability of the results. Second, BCI-controlled hand robot interventions have significant technical characteristics, leading to a high risk of bias in the implementation of blinding. Third, the MAS (finger flexor) is an ordinal scale with non-uniform intervals between categories (0, 1, 1+, 2, 3, and 4). Analyzing it as a continuous variable may lack methodological justification; therefore, the findings regarding spasticity reduction should be interpreted as exploratory. Fourth, long-term follow-up data were limited in the included studies, which restricted the evaluation of the sustained effects of BCI-controlled hand robot training. Fifth, several subgroup analyses in the present study were based on only a single included study, including those related to robot type (end-effector and soft robotic glove subgroups), BCI paradigm (SSVEP-BCI and MA-BCI subgroups), impairment severity (severe upper limb impairment subgroup), and follow-up duration (6-, 12-, and 18-week follow-ups). Therefore, these findings may be statistically unstable and should be interpreted with caution. At present, such results should be considered exploratory rather than conclusive. In addition, because the literature search was completed on 13 March 2026, studies published after this date were not included in the present review.

## 6. Conclusions

This meta-analysis shows that hand robot training controlled by BCI significantly improves overall upper limb motor function and proximal motor function in post-stroke patients. Compared with conventional rehabilitation, BCI-controlled hand robot training shows more significant efficacy in subacute patients, but has limited improvement in patients with severe upper limb dysfunction. Compared with hand robot training alone, BCI-controlled hand robot training demonstrates no additional advantages in distal motor function, but shows better effects in reducing finger flexor spasticity. The above results indicate that BCI-controlled hand robot training has certain potential in post-stroke upper limb rehabilitation, and its advantages primarily concentrate on specific functional dimensions and patient subgroups. Future high-quality studies with larger sample sizes and long-term follow-up are still needed to further clarify its optimal target population and long-term efficacy.

## Figures and Tables

**Table 1:** Characteristics of participants in the included studies.

Study	*n* (M/F)	Age (Years) (Mean ± SD)	Disease Duration (Mean ± SD)	Stroke Stage	Upper Limb Impairment Severity
Ang K K 2014 [19]	EG: 4/2 CG1: 6/2 CG2: 4/3	EG: 54.0 ± 8.9 CG1: 51.1 ± 6.3 CG2: 58.0 ± 19.3	EG: 285.7 ± 64.0 (days) CG1: 398.2 ± 150.9 CG2: 455.4 ± 109.6	Chronic	moderate to severe impairment of upper extremity function (FMMA score 10–50)
Cantillo-Negrete J 2021 [31]	EG: 5/5 CG: 5/5	EG: 59.9 ± 12.8 CG: 59.9 ± 12.8	EG: 140 ± 83 (days) CG: 140 ± 83	Subacute: 7 Chronic: 3	with severe upper limb impairment
Cantillo-Negrete J 2025 [32]	EG: 6/4 CG: 8/1	EG: 47.80 ± 15.74 CG: 55.78 ± 14.96	EG: 12.68 ± 5.81 CG: 10.33 ± 6.26	Subacute: 6 Chronic: 13	hand paresis (Motricity index from 0 to 22)
Cheng N 2020 [12]	EG: 3/2 CG: 2/3	EG: 62.4 ± 4.7 CG: 61.4 ± 4.5	EG: 476.8 ± 302.0 (days) CG: 890.2 ± 257.23	Chronic	FMA scores of 10–45
Frolov A A 2017 [15]	EG: 34/21 CG: 14/5	EG: 57.76 ± 12.66 CG: 59.46 ± 9.92	EG: 267.20 ± 177.24 (days) CG: 226.80 ± 104.26	Subacute: 34 Chronic: 40	with severe upper limb paralysis
Guo N 2022 [33]	EG: 9/1 CG1: 8/2 CG2: 8/2	EG: 60.2 ± 9.3 CG1: 56.9 ± 6.1 CG2: 53.5 ± 8.3	EG: 12.5 ± 7.1 (months) CG1: 11.7 ± 5.4 CG2: 10.9 ± 7.9	Chronic	moderate to severe motor impairments of upper limb (FMA-UL between 5 and 50)
Ji X 2025 [18]	EG: 12/8 CG: 15/4	EG: 61.75 ± 10.35 CG: 60.05 ± 14.35	EG: 49.50 ± 23.36 (days) CG: 42.42 ± 24.50	Subacute	upper-limb/hand dysfunction (Brunnstrom hand stages II–IV)
Wu Q 2020 [34]	EG: 9/5 CG: 9/2	EG: 62.93 ± 10.56 CG: 64.82 ± 7.22	EG: 2.11 ± 0.30 (months) CG: 2.27 ± 0.98	Subacute	moderate to severe UL paralysis
Zanona A F 2023 [35]	EG: 12/11 CG: 11/10	EG: 62.2 ± 9.8 CG: 61 ± 3	EG: 13.9 ± 6 (months) CG: 12.5 ± 6.7	Subacute	motor impairment in the upper limb (FMA motor domain 10–60)
Liu M Y 2023 [36]	EG: 15/4 CG: 10/8	EG: 51.26 ± 11.06 CG: 52.89 ± 13.07	EG: 98.26 ± 48.25 (days) CG: 90.28 ± 52.15	Subacute	Brunnstrom stage ≥ II
Fu J 2023 [37]	EG: 23/7 CG: 25/6	EG: 55.93 ± 11.05 CG: 59.00 ± 14.49	EG: 95.50 ± 110.55 (days) CG: 83.67 ± 88.28	NR	Brunnstrom stages I–V (ranging from severe to moderate impairment); distribution not reported

Note: CG control group, EG experimental group, F female, FMA-UE Fugl-Meyer Assessment for Upper Extremity, M male, *n* sample size, SD standard deviation, NR not reported. In addition, Frolov et al. (2017) [15] used an unequal allocation design, with 55 patients in the BCI group and 19 patients in the control group.

**Table 2:** Characteristics of interventions in the included studies.

Study	BCI Type	Robot Type	EG Intervention	CG Intervention	Dose-Matched Intervention Amount	Outcome Time Points	Outcomes
Ang K K 2014 [19]	MI-BCI	Haptic knob robot	BCI coupled with Haptic Knob robot (60 min) + therapist-assisted arm mobilization (30 min)	CG1: Haptic Knob robot (60 min) + therapist-assisted arm mobilization (30 min) CG2: therapist-assisted arm mobilization (90 min)	90 min/session, 3 sessions/week, 6 weeks	Baseline Post-intervention (6 weeks) Follow-up (6, 18 weeks)	FMA-UE FMA-UE proximal FMA-UE distal
Cantillo-Negrete J 2021 [31]	MI-BCI	Robotic hand orthosis	BCI coupled with robotic hand orthosis	Conventional upper limb therapy (neurofacilitation, stretching, grip, strength, coordination training)	30–40 min/session, 3 sessions/week, 4 weeks	Baseline Post-intervention (4 weeks)	FMA-UE ARAT
Cantillo-Negrete J 2025 [32]	MI-BCI	Robotic hand orthosis	BCI-controlled robotic hand orthosis	Robotic hand orthosis with sham-BCI	5 sessions/week, 6 weeks, 30 sessions	Baseline Post-intervention (6 weeks) Follow-up (18 weeks)	FMA-UE ARAT
Cheng N 2020 [12]	MI-BCI	Soft robotic glove	BCI-controlled soft robotic glove (90 min) + standard arm therapy (30 min)	Soft robotic glove (90 min) + standard arm therapy (30 min)	120 min/session 3 sessions/week, 6 weeks	Baseline Post-intervention (6 weeks) Follow-up (6, 18 weeks)	FMA-UE ARAT
Frolov A A 2017 [15]	MI-BCI	Hand exoskeleton robot	BCI-controlled hand exoskeleton robot	Hand exoskeleton robot	30 min/session, 10 sessions, total 5 h	Baseline Post-intervention (after 10 sessions)	FMA-UE ARAT FMA-UE proximal FMA-UE distal
Guo N 2022 [33]	SSVEP-BCI	Soft robotic glove	SSVEP-BCI-controlled soft robotic glove	CG1: Soft robotic glove CG2: Conventional therapy	60 min/session 5 sessions/week, 2 weeks	Baseline Post-intervention (2 weeks) Follow-up (12 weeks)	FMA-UE FMA-UE proximal FMA-UE distal MAS-finger
Ji X 2025 [18]	MI-BCI	Soft robotic glove	BCI-controlled soft robotic glove (20 min) + conventional rehabilitation (60 min/day)	Soft robotic glove (20 min) + conventional rehabilitation (60 min/day)	80 min/session 5 sessions/week 4 weeks	Baseline Post-intervention (4 weeks)	FMA-UE ARAT
Wu Q 2020 [34]	MI-BCI	Hand exoskeleton robot	BCI-controlled hand exoskeleton robot (1 h/day) + conventional rehabilitation (1 h/day)	Conventional rehabilitation (2 h/day)	2 h/day 5 days/week 4 weeks	Baseline Post-intervention (4 weeks)	FMA-UE ARAT
Zanona A F 2023 [35]	MI-BCI	Hand exoskeleton robot	BCI-controlled exoskeleton hand (30 min/session) + Conventional therapy (50 min/session)	Conventional therapy (80 min/session)	80 min/session 5 sessions/week, 2 weeks	Baseline Post-intervention (2 weeks)	FMA-UE
Liu M Y 2023 [36]	MI-BCI	Hand rehabilitation robot	Motor imagery-based BCI training (30 min/day) + routine comprehensive rehabilitation (60 min/day)	Hand rehabilitation robot training (30 min/day) + routine comprehensive rehabilitation (60 min/day)	90 min/day, 5 days/week, 4 weeks	Baseline Post-intervention (4 weeks)	FMA-UE FMA-UE distal MAS-finger
Fu J 2023 [37]	MA-BCI	Hand exoskeleton robot	BCI-controlled hand exoskeleton training (grasp/open motor training)	Task-oriented guidance training	30 min/day, 5 days/week, 4 weeks	Baseline Post-intervention (4 weeks)	FMA-UE

Note: ARAT Action Research Arm Test, BCI brain-computer interface, CG control group, EG experimental group, FMA-UE Fugl-Meyer Assessment for Upper Extremity, MAS-finger Modified Ashworth Scale score for finger flexors, MI-BCI motor imagery-based brain-computer interface, SSVEP-BCI steady-state visual evoked potential-based brain-computer interface.

## Data Availability

All data generated or analyzed during this study are included in the article/Supplementary Materials.

## Supplementary Materials

The following supporting information can be downloaded at: https://www.mdpi.com/article/10.3390/brainsci16060552/s1, Table S1: PRISMA 2020 Checklist; Table S2: Search strategies; Table S3: Summary of reasons for exclusion of retrieved records; Table S4. GRADE assessment of the certainty of evidence for primary and secondary outcomes; Supplementary Figure S1. Forest plot of FMA-UE: BCI-robot vs. robot. Supplementary Figure S2. Forest plot of FMA-UE: BCI-robot vs. conventional rehabilitation. Supplementary Figure S3. Forest plot of ARAT: BCI-robot vs. robot. Supplementary Figure S4. Forest plot of ARAT: BCI-robot vs. conventional rehabilitation. Supplementary Figure S5. Forest plot of FMA-UE proximal score: BCI-robot vs. robot. Supplementary Figure S6. Forest plot of FMA-UE proximal score: BCI-robot vs. conventional rehabilitation. Supplementary Figure S7. Forest plot of FMA-UE distal score: BCI-robot vs. robot. Supplementary Figure S8. Forest plot of FMA-UE distal score: BCI-robot vs. conventional rehabilitation. Supplementary Figure S9. Forest plot of MAS finger flexor score: BCI-robot vs. robot. Supplementary Figure S10. Forest plot of subgroup analysis by stroke stage for FMA-UE: BCI-robot vs. robot. Supplementary Figure S11. Forest plot of subgroup analysis by stroke stage for FMA-UE: BCI-robot vs. conventional rehabilitation. Supplementary Figure S12. Forest plot of subgroup analysis by stroke stage for ARAT: BCI-robot vs. robot. Supplementary Figure S13. Forest plot of subgroup analysis by robot type for FMA-UE: BCI-robot vs. robot. Supplementary Figure S14. Forest plot of subgroup analysis by robot type for FMA-UE: BCI-robot vs. conventional rehabilitation. Supplementary Figure S15. Forest plot of subgroup analysis by robot type for ARAT: BCI-robot vs. robot. Supplementary Figure S16. Forest plot of subgroup analysis by BCI paradigm for FMA-UE: BCI-robot vs. robot. Supplementary Figure S17. Forest plot of subgroup analysis by BCI paradigm for FMA-UE: BCI-robot vs. conventional rehabilitation. Supplementary Figure S18. Forest plot of subgroup analysis by upper limb impairment severity for FMA-UE: BCI-robot vs. robot. Supplementary Figure S19. Forest plot of subgroup analysis by upper limb impairment severity for FMA-UE: BCI-robot vs. conventional rehabilitation. Supplementary Figure S20. Forest plot of subgroup analysis by upper limb impairment severity for ARAT: BCI-robot vs. robot. Supplementary Figure S21. Forest plot of subgroup analysis by follow-up duration for FMA-UE: BCI-robot vs. robot. Supplementary Figure S22. Forest plot of subgroup analysis by follow-up duration for FMA-UE: BCI-robot vs. conventional rehabilitation. Supplementary Figure S23. Forest plot of subgroup analysis by follow-up duration for ARAT: BCI-robot vs. robot.

## Abbreviations

The following abbreviations are used in this manuscript:

ARAT	Action Research Arm Test
BCI	Brain-computer interface
CI	Confidence interval
CG	Control group
EG	Experimental group
FMA-UE	Fugl-Meyer Assessment for Upper Extremity
MAS	Modified Ashworth Scale
MD	Mean difference
MI-BCI	Motor imagery-based brain-computer interface
RCT	Randomized controlled trial
SD	Standard deviation
SSVEP	Steady-state visual evoked potential
