# Supplementary material for: Effects of Brain-Computer Interface-Controlled Hand Robot Training on Post-Stroke Recovery of Upper Limb Motor Functions: A Meta-Analysis of Dose-Matched Randomized Controlled Trials
Source: Brain Sci. 2026 May 22;16(6):552. doi: 10.3390/brainsci16060552 (PMC13297567; doi:10.3390/brainsci16060552)
Supplement: Supplementary file 1 [file brainsci-16-00552-s001.zip › brainsci-4308945-supplementary.pdf]

Supplementary materials

Supplementary Table S1. PRISMA 2020 Checklist

Section and Topic	Item #	Checklist item	Location where item is reported
**TITLE**			
Title	1	Identify the report as a systematic review.	pg. 1
**ABSTRACT**			
Abstract	2	See the PRISMA 2020 for Abstracts checklist.	pg. 1
**INTRODUCTION**			
Rationale	3	Describe the rationale for the review in the context of existing knowledge.	pg. 2
Objectives	4	Provide an explicit statement of the objective(s) or question(s) the review addresses.	pg. 3
**METHODS**			
Eligibility criteria	5	Specify the inclusion and exclusion criteria for the review and how studies were grouped for the syntheses.	pg. 4
Information sources	6	Specify all databases, registers, websites, organisations, reference lists and other sources searched or consulted to identify studies. Specify the date when each source was last searched or consulted.	pg. 3-4
Search strategy	7	Present the full search strategies for all databases, registers and websites, including any filters and limits used.	pg. 3-4
Selection process	8	Specify the methods used to decide whether a study met the inclusion criteria of the review, including how many reviewers screened each record and each report retrieved, whether they worked independently, and if applicable, details of automation tools used in the process.	pg. 4
Data collection process	9	Specify the methods used to collect data from reports, including how many reviewers collected data from each report, whether they worked independently, any processes for obtaining or confirming data from study investigators, and if applicable, details of automation tools used in the process.	pg. 4
Data items	10a	List and define all outcomes for which data were sought. Specify whether all results that were compatible with each outcome domain in each study were sought (e.g. for all measures, time points, analyses), and if not, the methods used to decide which results to collect.	pg. 4-6
	10b	List and define all other variables for which data were sought (e.g. participant and intervention characteristics, funding sources). Describe any assumptions made about any missing or unclear information.	pg. 4
Study risk of bias assessment	11	Specify the methods used to assess risk of bias in the included studies, including details of the tool(s) used, how many reviewers assessed each study and whether they worked independently, and if applicable, details of automation tools used in the process.	pg. 4
Effect measures	12	Specify for each outcome the effect measure(s) (e.g. risk ratio, mean difference) used in the synthesis or presentation of results.	pg. 5
Synthesis methods	13a	Describe the processes used to decide which studies were eligible for each synthesis (e.g. tabulating the study intervention characteristics and comparing against the planned groups for each synthesis (item #5)).	pg. 4; pg. 6-9
	13b	Describe any methods required to prepare the data for presentation or synthesis, such as handling of missing summary statistics, or data conversions.	pg. 5
	13c	Describe any methods used to tabulate or visually display results of individual studies and syntheses.	pg. 4; pg.

			10-13
	13d	Describe any methods used to synthesize results and provide a rationale for the choice(s). If meta-analysis was performed, describe the model(s), method(s) to identify the presence and extent of statistical heterogeneity, and software package(s) used.	pg. 5
	13e	Describe any methods used to explore possible causes of heterogeneity among study results (e.g. subgroup analysis, meta-regression).	pg. 5
	13f	Describe any sensitivity analyses conducted to assess robustness of the synthesized results.	pg. 5
Reporting bias assessment	14	Describe any methods used to assess risk of bias due to missing results in a synthesis (arising from reporting biases).	pg. 5
Certainty assessment	15	Describe any methods used to assess certainty (or confidence) in the body of evidence for an outcome.	pg. 4
**RESULTS**			
Study selection	16a	Describe the results of the search and selection process, from the number of records identified in the search to the number of studies included in the review, ideally using a flow diagram.	pg. 6
	16b	Cite studies that might appear to meet the inclusion criteria, but which were excluded, and explain why they were excluded.	pg. 6
Study characteristics	17	Cite each included study and present its characteristics.	pg. 6-9
Risk of bias in studies	18	Present assessments of risk of bias for each included study.	pg. 9-10
Results of individual studies	19	For all outcomes, present, for each study: (a) summary statistics for each group (where appropriate) and (b) an effect estimate and its precision (e.g. confidence/credible interval), ideally using structured tables or plots.	pg. 10-13
Results of syntheses	20a	For each synthesis, briefly summarise the characteristics and risk of bias among contributing studies.	pg. 10-13
	20b	Present results of all statistical syntheses conducted. If meta-analysis was done, present for each the summary estimate and its precision (e.g. confidence/credible interval) and measures of statistical heterogeneity. If comparing groups, describe the direction of the effect.	pg. 10-13
	20c	Present results of all investigations of possible causes of heterogeneity among study results.	pg. 11-13
	20d	Present results of all sensitivity analyses conducted to assess the robustness of the synthesized results.	pg. 10
Reporting biases	21	Present assessments of risk of bias due to missing results (arising from reporting biases) for each synthesis assessed.	pg. 5
Certainty of evidence	22	Present assessments of certainty (or confidence) in the body of evidence for each outcome assessed.	pg. 13-14
**DISCUSSION**			
Discussion	23a	Provide a general interpretation of the results in the context of other evidence.	pg. 14-18
	23b	Discuss any limitations of the evidence included in the review.	pg.18
	23c	Discuss any limitations of the review processes used.	pg.18
	23d	Discuss implications of the results for practice, policy, and future research.	pg.18
**OTHER INFORMATION**			
Registration and protocol	24a	Provide registration information for the review, including register name and registration number, or state that the review was not registered.	pg. 3

	24b	Indicate where the review protocol can be accessed, or state that a protocol was not prepared.	pg. 3
	24c	Describe and explain any amendments to information provided at registration or in the protocol.	
Support	25	Describe sources of financial or non-financial support for the review, and the role of the funders or sponsors in the review.	pg. 19
Competing interests	26	Declare any competing interests of review authors.	pg. 19
Availability of data, code and other materials	27	Report which of the following are publicly available and where they can be found: template data collection forms; data extracted from included studies; data used for all analyses; analytic code; any other materials used in the review.	pg. 19

Supplementary Table S2. Search strategies

PubMed（n=360）
(("Robotics"[Mesh] OR rehabilitation robot OR rehabilitation robotics OR robotic rehabilitation OR robot-assisted rehabilitation OR robot-assisted therapy OR robot-assisted training OR robotic therapy OR robotic training OR robotic device OR robotic system OR robotic orthosis OR robotic glove OR robotic exoskeleton OR upper limb robot OR hand rehabilitation robot OR robotic hand OR hand robot OR robotic arm OR robot-assisted upper limb therapy OR exoskeleton robot OR upper limb exoskeleton OR hand exoskeleton OR robotic hand rehabilitation OR hand robotic therapy) AND ("Brain-Computer Interfaces"[Mesh] OR brain-computer interface OR brain computer interface OR BCI OR brain-machine interface OR brain machine interface OR BMI OR EEG-based BCI OR EEG BCI OR motor imagery BCI OR MI-BCI OR BCI-based rehabilitation OR BCI-based training OR BCI-assisted rehabilitation OR BCI-controlled rehabilitation OR BCI-controlled robot OR brain-computer interface system OR brain-computer interface training OR SSVEP-BCI OR P300-BCI) AND ("Stroke"[Mesh] OR Strokes OR Cerebrovascular Accident OR Cerebrovascular Accidents OR Cerebral Stroke OR Cerebral Strokes OR Stroke, Cerebral OR Strokes, Cerebral OR Cerebrovascular Apoplexy OR Apoplexy, Cerebrovascular OR Vascular Accident, Brain OR Brain Vascular Accident OR Brain Vascular Accidents OR Vascular Accidents, Brain OR Cerebrovascular Stroke OR Cerebrovascular Strokes OR Stroke, Cerebrovascular OR Strokes, Cerebrovascular OR Apoplexy OR CVA (Cerebrovascular Accident) OR CVAs (Cerebrovascular Accident) OR Stroke, Acute OR Acute Stroke OR Acute Strokes OR Strokes, Acute OR Cerebrovascular Accident, Acute OR Acute Cerebrovascular Accident OR Acute Cerebrovascular Accidents OR Cerebrovascular Accidents, Acute))
**Web of Science = (530)**
#1 ((((((((((((((((((((((TS=(Robotics)) OR TS=(rehabilitation robot)) OR TS=(rehabilitation robotics)) OR TS=(robotic rehabilitation)) OR TS=(robot-assisted rehabilitation)) OR TS=(robot-assisted therapy)) OR TS=(robot-assisted training)) OR TS=(robotic therapy)) OR TS=(robotic training)) OR TS=(robotic device)) OR TS=(robotic system)) OR TS=(robotic orthosis)) OR TS=(robotic glove)) OR TS=(robotic exoskeleton)) OR TS=(upper limb robot)) OR TS=(hand rehabilitation robot)) OR

TS=(robotic hand)) OR TS=(hand robot)) OR TS=(robotic arm)) OR TS=(robot-assisted upper limb therapy)) OR TS=(exoskeleton robot)) OR TS=(upper limb exoskeleton)) OR TS=(hand exoskeleton)) OR TS=(robotic hand rehabilitation)) OR TS=(hand robotic therapy))

#2 ((((((((((((((((((((TS=(Brain-Computer Interface)) OR TS=(brain-computer interface)) OR TS=(brain computer interface)) OR TS=(BCI)) OR TS=(brain-machine interface)) OR TS=(brain machine interface)) OR TS=(BMI)) OR TS=(EEG-based BCI)) OR TS=(EEG BCI)) OR TS=(motor imagery BCI)) OR TS=(MI-BCI)) OR TS=(BCI-based rehabilitation)) OR TS=(BCI-based training)) OR TS=(BCI-assisted rehabilitation)) OR TS=(BCI-controlled rehabilitation)) OR TS=(BCI-controlled robot)) OR TS=(brain-computer interface system)) OR TS=(brain-computer interface training)) OR TS=(SSVEP-BCI)) OR TS=(P300-BCI))

#3 (((((((((((((((((((((((((((((TS=(Stroke)) OR TS=(Strokes)) OR TS=(Cerebrovascular Accident)) OR TS=(Cerebrovascular Accidents)) OR TS=(Cerebral Stroke)) OR TS=(Cerebral Strokes)) OR TS=(Stroke, Cerebral)) OR TS=(Strokes, Cerebral)) OR TS=(Cerebrovascular Apoplexy)) OR TS=(Apoplexy, Cerebrovascular)) OR TS=(Vascular Accident, Brain)) OR TS=(Brain Vascular Accident)) OR TS=(Brain Vascular Accidents)) OR TS=(Vascular Accidents, Brain)) OR TS=(Cerebrovascular Stroke)) OR TS=(Cerebrovascular Strokes)) OR TS=(Stroke, Cerebrovascular)) OR TS=(Strokes, Cerebrovascular)) OR TS=(Apoplexy)) OR TS=(CVA (Cerebrovascular Accident))) OR TS=(CVAs (Cerebrovascular Accident))) OR TS=(Stroke, Acute)) OR TS=(Acute Stroke)) OR TS=(Acute Strokes)) OR TS=(Strokes, Acute)) OR TS=(Cerebrovascular Accident, Acute)) OR TS=(Acute Cerebrovascular Accident)) OR TS=(Acute Cerebrovascular Accidents)) OR TS=(Cerebrovascular Accidents, Acute))

#4 #1 AND #2 AND #3

Embase = (n=94)

#1 'robotics'/exp

#2 'rehabilitation robot':ab,ti OR 'rehabilitation robotics':ab,ti OR 'robotic rehabilitation':ab,ti OR 'robot-assisted rehabilit ation':ab,ti OR 'robot-assisted therapy':ab,ti OR 'robot-assisted training':ab,ti OR 'robotic therapy':ab,ti OR 'robotic traini ng':ab,ti OR 'robotic device':ab,ti OR 'robotic system':ab,ti OR 'robotic orthosis':ab,ti OR 'robotic glove':ab,ti OR 'robot ic exoskeleton':ab,ti OR 'upper limb robot':ab,ti OR 'hand rehabilitation robot':ab,ti OR 'robotic hand':ab,ti OR 'hand r obot':ab,ti OR 'robotic arm':ab,ti OR 'robot-assisted upper limb therapy':ab,ti OR 'exoskeleton robot':ab,ti OR 'upper li mb exoskeleton':ab,ti OR 'hand exoskeleton':ab,ti OR 'robotic hand rehabilitation':ab,ti OR 'hand robotic therapy':ab,ti

#3 #1 OR #2

#4 'brain computer interface'/exp

#5 'brain-computer interface':ab,ti OR 'brain computer interface':ab,ti OR 'bci':ab,ti OR 'brain-machine interface':ab,ti O R 'brain machine interface':ab,ti OR 'bmi':ab,ti OR 'eeg-based bci':ab,ti OR 'eeg bci':ab,ti OR 'motor imagery bci':ab,ti OR 'mi-bci':ab,ti OR 'bci-based rehabilitation':ab,ti OR 'bci-based training':ab,ti OR 'bci-assisted rehabilitation':ab,ti OR

'bci-controlled rehabilitation':ab,ti OR 'bci-controlled robot':ab,ti OR 'brain-computer interface system':ab,ti OR 'brain-co mputer interface training':ab,ti OR 'ssvep-bci':ab,ti OR 'p300-bci':ab,ti<br>#6 #4 OR #5<br>#7 'stroke patient'/exp<br>#8 strokes:ab,ti OR 'cerebrovascular accident':ab,ti OR 'cerebrovascular accidents':ab,ti OR 'cerebral stroke':ab,ti OR 'c erebral strokes':ab,ti OR 'stroke, cerebral':ab,ti OR 'strokes, cerebral':ab,ti OR 'cerebrovascular apoplexy':ab,ti OR 'apopl exy, cerebrovascular':ab,ti OR 'vascular accident, brain':ab,ti OR 'brain vascular accident':ab,ti OR 'brain vascular accid ents':ab,ti OR 'vascular accidents, brain':ab,ti OR 'cerebrovascular stroke':ab,ti OR 'cerebrovascular strokes':ab,ti OR 'str oke, cerebrovascular':ab,ti OR 'strokes, cerebrovascular':ab,ti OR apoplexy:ab,ti OR (cva:ab,ti AND 'cerebrovascular acc ident':ab,ti) OR (cvas:ab,ti AND 'cerebrovascular accident':ab,ti) OR 'stroke, acute':ab,ti OR 'acute stroke':ab,ti OR 'acu te strokes':ab,ti OR 'strokes, acute':ab,ti OR 'cerebrovascular accident, acute':ab,ti OR 'acute cerebrovascular accident':a b,ti OR 'acute cerebrovascular accidents':ab,ti OR 'cerebrovascular accidents, acute':kw<br>#9 #7 OR #8<br>#10 #3 AND #6 AND #9
**Cochrane library =(n=57)**
#1 MeSH descriptor: [Robotics] explode all trees<br>#2 (rehabilitation robot):ti,ab,kw OR (rehabilitation robotics):ti,ab,kw OR (robotic rehabilitation):ti,ab,kw OR (robot-assisted rehabilitation):ti,ab,kw OR (robot-assisted therapy):ti,ab,kw OR (robot-assisted training):ti,ab,kw OR (robotic therapy):ti,ab,kw OR (robotic training):ti,ab,kw OR (robotic device):ti,ab,kw OR (robotic system):ti,ab,kw OR (robotic orthosis):ti,ab,kw OR (robotic glove):ti,ab,kw OR (robotic exoskeleton):ti,ab,kw OR (upper limb robot):ti,ab,kw OR (hand rehabilitation robot):ti,ab,kw OR (robotic hand):ti,ab,kw OR (hand robot):ti,ab,kw OR (robotic arm):ti,ab,kw OR (robot-assisted upper limb therapy):ti,ab,kw OR (exoskeleton robot):ti,ab,kw OR (upper limb exoskeleton):ti,ab,kw OR (hand exoskeleton):ti,ab,kw OR (robotic hand rehabilitation):ti,ab,kw OR (hand robotic therapy):ti,ab,kw<br>#3 #1 OR #2<br>#4 MeSH descriptor: [Brain-Computer Interfaces] explode all trees<br>#5 (brain-computer interface):ti,ab,kw OR (brain computer interface):ti,ab,kw OR (BCI):ti,ab,kw OR (brain-machine interface):ti,ab,kw OR (brain machine interface):ti,ab,kw OR (BMI):ti,ab,kw OR (EEG-based BCI):ti,ab,kw OR (EEG BCI):ti,ab,kw OR (motor imagery BCI):ti,ab,kw OR (MI-BCI):ti,ab,kw OR (BCI-based rehabilitation):ti,ab,kw OR (BCI-based training):ti,ab,kw OR (BCI-assisted rehabilitation):ti,ab,kw OR (BCI-controlled rehabilitation):ti,ab,kw OR (BCI-controlled robot):ti,ab,kw OR (brain-computer interface system):ti,ab,kw OR (brain-computer interface training):ti,ab,kw OR (SSVEP-BCI):ti,ab,kw

#6 #4 OR #5<br>#7 MeSH descriptor: [Stroke] explode all trees<br>#8 (Strokes):ti,ab,kw OR (Cerebrovascular Accident):ti,ab,kw OR (Cerebrovascular Accidents):ti,ab,kw OR (Cerebral Stroke):ti,ab,kw OR (Cerebral Strokes):ti,ab,kw OR (Stroke, Cerebral):ti,ab,kw OR (Strokes, Cerebral):ti,ab,kw OR (Cerebrovascular Apoplexy):ti,ab,kw OR (Apoplexy, Cerebrovascular):ti,ab,kw OR (Vascular Accident, Brain):ti,ab,kw OR (Brain Vascular Accident):ti,ab,kw OR (Brain Vascular Accidents):ti,ab,kw OR (Vascular Accidents, Brain):ti,ab,kw OR (Cerebrovascular Stroke):ti,ab,kw OR (Cerebrovascular Strokes):ti,ab,kw OR (Stroke, Cerebrovascular):ti,ab,kw OR (Strokes, Cerebrovascular):ti,ab,kw OR (Apoplexy):ti,ab,kw OR (CVA (Cerebrovascular Accident)):ti,ab,kw OR (CVAs (Cerebrovascular Accident)):ti,ab,kw OR (Stroke, Acute):ti,ab,kw OR (Acute Stroke):ti,ab,kw OR (Acute Strokes):ti,ab,kw OR (Strokes, Acute):ti,ab,kw OR (Cerebrovascular Accident, Acute):ti,ab,kw OR (Acute Cerebrovascular Accident):ti,ab,kw OR (Acute Cerebrovascular Accidents):ti,ab,kw OR (Cerebrovascular Accidents, Acute):ti,ab,kw<br>#9 #7 OR #8<br>#10 #3 AND #6 AND #9
**CNKI（62）**
（主题：康复机器人）OR（主题：机器人康复）OR（主题：机器人辅助康复）OR（主题：机器人辅助治疗）OR（主题：外骨骼机器人）OR（主题：上肢康复机器人）OR（主题：手部康复机器人）OR（主题：机器人手套）OR（主题：上肢外骨骼）AND（主题：脑机接口）OR（主题：脑-机接口）OR（主题：BCI）OR（主题：运动想象脑机接口）OR（主题：MI-BCI）OR（主题：脑机接口训练）OR（主题：脑机接口康复）OR（主题：EEG 脑机接口）OR（主题：SSVEP-BCI）OR（主题：P300-BCI）AND（主题：卒中）OR（主题：中风）OR（主题：脑卒中）OR（主题：脑血管意外）OR（主题：脑血管病）OR（主题：急性卒中）OR（主题：脑梗死）OR（主题：脑出血）OR（主题：急性脑卒中）
**Sinomed（20）**
(("卒中"[常用字段:智能] OR "中风"[常用字段:智能] OR "脑卒中"[常用字段:智能] OR "脑血管意外"[常用字段:智能] OR "脑血管病"[常用字段:智能] OR "急性卒中"[常用字段:智能] OR "脑梗死"[常用字段:智能] OR "脑出血"[常用字段:智能] OR "急性脑卒中"[常用字段:智能])) AND (("脑机接口"[常用字段:智能] OR "脑-机接口"[常用字段:智能] OR "BCI"[常用字段:智能] OR "运动想象脑机接口"[常用字段:智能] OR "MI-BCI"[常用字段:智能] OR "脑机接口训练"[常用字段:智能] OR "脑机接口康复"[常用字段:智能] OR "EEG 脑机接口"[常用字段:智能] OR "SSVEP-BCI"[常用字段:智能] OR "P300-BCI"[常用字段:智能])) AND (("康复机器人"[常用字段:智能] OR "机器人康复"[常用字段:智能] OR "机器人辅助康复"[常用字段:智能] OR "机器人辅助治疗"[常用字段:智能] OR "外骨骼机器人"[常用字段:智能] OR "上肢康复机器人"[常用字段:智能] OR "手部康复机器人"[常用字段:智能] OR "机器人手套"[常用字段:智能] OR "上肢外骨骼"[常用字段:智能]))
**Wanfang Data（29）**

题名或关键词:(康复机器人 or 机器人康复 or 机器人辅助康复 or 机器人辅助治疗 or 外骨骼机器人 or 上肢康复机器人 or 手部康复机器人 or 机器人手套 or 上肢外骨骼) and 题 名或关键词:(脑机接口 or 脑-机接口 or BCI or 运动想象脑机接口 or MI-BCI or 脑机接口训练 or 脑机接口康复 or EEG 脑机接口 or SSVEP-BCI or P300-BCI) and 题名或关键词:(卒中 or 中风 or 脑卒中 or 脑血管意外 or 脑血管病 or 急性卒中 or 脑梗死 or 脑出血 or 急性脑卒中)
**VIP Database（547）**
题名或关键词=康复机器人 OR 机器人康复 OR 机器人辅助康复 OR 机器人辅助治疗 OR 外骨骼机器人 OR 上肢康复机器人 OR 手部康复机器人 OR 机器人手套 OR 上肢外骨骼 AND 题名或关键词=脑机接口 OR 脑-机接口 OR BCI OR 运动想象脑机接口 OR MI-BCI OR 脑机接口训练 OR 脑机接口康复 OR EEG 脑机接口 OR SSVEP-BCI OR P300-BCI AND 题名或关键词=卒中 OR 中风 OR 脑卒中 OR 脑血管意外 OR 脑血管病 OR 急性卒中 OR 脑梗死 OR 脑出血 OR 急性脑卒中

Supplementary Table S3 Summary of reasons for exclusion of retrieved records (n = 69)

No.	Study	Reason for exclusion
**Reasons for exclusion of database records (n = 64)**		
1	Neural activity modulations and motor recovery following brain-exoskeleton interface mediated stroke rehabilitation	Not RCT (single-arm trial)
2	Contralesional Brain-Computer Interface Control of a Powered Exoskeleton for Motor Recovery in Chronic Stroke Survivors	
3	Can Corticomuscular Coupling be Useful in Designing Hybrid-Brain Robot Interfaces Towards Hand Functional Recovery?	
4	Active Physical Practice Followed by Men a Practice Using BCI-Driven Hand Exoskeleton: A Pilot Trial for Clinical Effectiveness and Usability	
5	Changes in electroencephalography complexity and functional magnetic resonance imaging connectivity following robotic hand training in chronic stroke	
6	Recovery Dynamics in Patients with Poststroke Motor Disorders after Multiple Courses of Neurorehabilitation Using an Exoskeleton Controlled by a Brain–Computer Interface	
7	Modulation of Functional Connectivity and Low-Frequency Fluctuations After Brain-Computer Interface-Guided Robot Hand Training in Chronic Stroke: A 6-Month Follow-Up Study	
8	Brain-Computer Interface-Robot Training Enhances Upper Extremity Performance and Changes the Cortical Activation in Stroke Patients: A Functional Near-Infrared Spectroscopy	

	Study	
9	Exploring neural activity changes during motor imagery-based brain-computer interface training with robotic hand for upper limb rehabilitation in ischemic stroke patients: a pilot study	
10	Exploring neural activity changes during motor imagery-based brain-computer interface training with robotic hand for upper limb rehabilitation in ischemic stroke patients: a pilot study	
11	Controlling pre-movement sensorimotor rhythm can improve finger extension after stroke	
12	ACTIVATION OF SENSORIMOTOR INTEGRATION PROCESSES WITH A BRAIN-COMPUTER INTERFACE	
13	A hybrid brain-muscle-machine interface for stroke rehabilitation: Usability and functionality validation in a 2-week intensive intervention	
14	Interhemispheric Functional Reorganization and its Structural Base After BCI-Guided Upper-Limb Training in Chronic Stroke	
15	Novel personalized treatment strategy for patients with chronic stroke with severe upper-extremity impairment: The first patient of the AVANCER trial	Not RCT (case report)
16	Combination of Brain-Computer Interface Training and Goal-Directed Physical Therapy in Chronic Stroke: A Case Report	
17	Application of a Brain-Computer Interface System with Visual and Motor Feedback in Limb and Brain Functional Rehabilitation after Stroke: Case Report	
18	Training cortical signals by means of a BMI-EEG system, its evolution and intervention. A case report	
19	Brain–Computer Interfaces in Poststroke Rehabilitation: a Clinical Neuropsychological Study	Secondary analysis
20	Brain-computer interface-based (BCI) based arm robotic rehabilitation for stroke: a feasibility study and randomized controlled trial	Conference abstract
21	Brain computer interface (BCI) based robotic rehabilitation for the stroke upper limb	
22	Brain computer interface based robotic rehabilitation for upper limb hemiplegia following stroke	
23	Clinical studies and neuroimage analysis of brain-computer inerface for stroke rehabilitation	
24	BCI-Based Dexterous Hand Rehabilitation Robot for Grasping Training of Post Stroke	
25	Brain computer interface for post-stroke rehabilitation of upper-limb function: Results of	

	randomized control trial	
26	Botulinum toxin A therapy of post-stroke hand spasticity in combination with brain-computer interface plus exoskeleton	
27	Engineered Devices to Support Stroke Rehabilitation	
28	A Brain-Computer Interface for rehabilitation after stroke	
29	Improving of the Effectiveness of Motor-Imagery Training With BCI Technology in Hand Exoskeleton in Post-Stroke Rehabilitation	
30	A Hybrid Brain-Machine Interface based on EEG and EMG activity for the Motor Rehabilitation of Stroke Patients	
31	An EEG-Based Brain-Machine Interface to Control a 7-Degrees of Freedom Exoskeleton for Stroke Rehabilitation	
32	Preliminary Results from a Stroke Rehabilitation Protocol Utilizing a Robotic BMI-Exoskeleton System	
33	A Synchronous and Closed-Loop Architecture of BCI-Based Rehabilitation System for Stroke with Robot and Virtual Reality	
34	Personalized upper limb stroke rehabilitation using data-driven multi-modal electroencephalography (EEG) and near-infrared spectroscopy (NIRS) based brain computer interface (BCI) with soft robotic glove	
35	Comprehensive Evaluation of Stroke Rehabilitation Dynamics: Integrating Brain-Computer Interface with Robotized Orthesic Hand and Longitudinal EEG Changes	
36	Combining Soft Robotics and Brain-Machine Interfaces for Stroke Rehabilitation	
37	Assessing the impact of assistive hand exoskeletons on bimanual tasks in severe stroke with the Berlin Bimanual Test for Stroke (BeBiTS)	
38	脑机接口机器人联合任务导向性训练对卒中后手功能障碍影响的临床研究	Mismatched intervention dose
39	Development of a Brain-machine Interface for Stroke Rehabilitation Using Event-related Desynchronization and Proprioceptive Feedback	Relevant data unavailable
40	Facilitating effects of transcranial direct current stimulation on motor imagery brain-computer interface with robotic feedback for stroke rehabilitation	Ineligible intervention
41	Efficacy of brain-computer interface training with motor imagery-contingent feedback in improving upper limb function and neuroplasticity among persons with chronic stroke: a	

	double-blinded, parallel-group, randomized controlled trial	
42	New Artificial Intelligence-Integrated Electromyography-Driven Robot Hand for Upper Extremity Rehabilitation of Patients With Stroke: A Randomized, Controlled Trial	
43	EEG-Based Brain Network Analysis of Chronic Stroke Patients After BCI Rehabilitation Training	
44	A Randomized Controlled Trial of EEG-Based Motor Imagery Brain-Computer Interface Robotic Rehabilitation for Stroke	
45	脑机接口联合上肢康复机器人对脑卒中患者上肢功能的影响	
46	Effects of a Brain-Computer Interface-Operated Lower Limb Rehabilitation Robot on Motor Function Recovery in Patients with Stroke	Retracted article
47	Ideomotor training combining the use with integrated application of electromyostimulation and a robotic brain-computer interface in post-stroke upper limb dysfunction: a randomized controlled trial	Full text unavailable
48	DYNAMICS OF RECOVERY IN PATIENTS WITH POST-STROKE MOVEMENT DISORDERS AFTER REPEATED COURSES OF NEUROREHABILITATION WITH THE USE OF THE "BRAIN-COMPUTER" INTERFACE AND EXOSKELETON	
49	脑机接口控制外骨骼机器人训练对脑卒中后上肢运动功能障碍的有效性研究	Master’s or doctoral thesis
50	脑控外肢体训练对脑卒中患者上肢及手功能障碍的影响研究	
51	Enhanced Brain Functional Interaction Following BCI-Guided Supernumerary Robotic Finger Training Based on Sixth-Finger Motor Imagery	Non-stroke population
52	Assisting drinking with an affordable BCI-controlled wearable robot and electrical stimulation: a preliminary investigation	
53	Reinforcement learning of self-regulated sensorimotor β-oscillations improves motor performance	
54	A Personalized Multimodal BCI-Soft Robotics System for Rehabilitating Upper Limb Function in Chronic Stroke Patients	
55	Optimization of machine learning method combined with brain-computer interface rehabilitation system	
56	EEG-modulated robotic rehabilitation system for upper extremity	
57	Hybrid BCI for upper limb rehabilitation: integrating MI with peripheral field SSVEP stimulation	

58	The Role of Vibrotactile Stimulation in Soft Rehabilitation Glove-Assisted Hand Rehabilitation Training: A Pilot Study	
59	Residual Upper Arm Motor Function Primes Innervation of Paretic Forearm Muscles in Chronic Stroke after Brain-Machine Interface (BMI) Training	No relevant outcome measures
60	Effect of Brain-Computer Interface-Controlled Ankle Robot Training on Post-Stroke Motor Rehabilitation and Resting QEEG Neuroplasticity: A Randomized Controlled Trial	
61	Effects of Training with a Brain–Computer Interface-Controlled Robot on Rehabilitation Outcome in Patients with Subacute Stroke: a Randomized Controlled Trial	
62	基于脑机交互技术的康复机器人对卒中后偏瘫康复治疗的临床研究	
63	脑-机接口控制下机器人辅助康复训练治疗亚急性期脑卒中患者效果的随机对照临床研究	
64	脑机接口外骨骼对卒中患者下肢康复疗效及皮层激活探究	
**Reasons for exclusion of other retrieved records (n = 5)**		
1	Brain-Machine Interface in Chronic Stroke: Randomized Trial Long-Term Follow-up	Non-RCT (long-term follow-up report)
2	Brain–Machine Interface in Chronic Stroke Rehabilitation: A Controlled Study	No relevant outcome measures
3	Resting State Changes in Functional Connectivity Correlate With Movement Recovery for BCI and Robot-Assisted Upper-Extremity Training After Stroke	Ineligible intervention
4	Preliminary results of a controlled study of BCI–exoskeleton technology efficacy in patients with poststroke arm paresis	Duplicate report
5	Differentiated Effects of Robot Hand Training With and Without Neural Guidance on Neuroplasticity Patterns in Chronic Stroke	Relevant data unavailable

Supplementary Table S4. GRADE assessment of the certainty of evidence for primary and secondary outcomes

Outcome	Comparison	Studies	Sample size	Effect size (MD, 95% CI)	I^2	Risk of bias	Inconsistency	Indirectness	Imprecision	Publication bias	Certainty of evidence
**FMA-UE**	BCI-robot vs. robot	7	213	4.87 (1.04, 8.69)	0%	-1	0	0	-1	0	Low (⊕⊕◯◯)
**FMA-UE**	BCI-robot vs. CR	6	183	6.55 (3.49, 9.61)	1%	-1	0	0	-1	0	Low (⊕⊕◯◯)
**ARAT**	BCI-robot vs. robot	4	142	1.87 (-4.01, 7.75)	0%	-1	0	0	-2	0	Very low (⊕◯◯◯)
**FMA-UE proximal**	BCI-robot vs. robot	3	108	4.44 (0.15, 8.74)	0%	-1	0	0	-1	0	Low (⊕⊕◯◯)

**FMA-UE proximal**	BCI-robot vs. CR	2	33	7.92(1.92–13.91)	0%	-1	0	0	-1	0	Low (⊕⊕◯◯)
**FMA-UE distal**	BCI-robot vs. robot	4	145	0.17 (-0.71, 1.04)	0%	-1	0	0	-1	0	Low (⊕⊕◯◯)
**FMA-UE distal**	BCI-robot vs. CR	2	33	5.06(1.05, 9.07)	0%	-1	0	0	-1	0	Low (⊕⊕◯◯)
**MAS-finger**	BCI-robot vs. robot	2	57	-0.44 (-0.68, -0.21)	0%	-1	0	-1	-1	0	Very low (⊕◯◯◯)

CR = conventional rehabilitation. GRADE certainty of evidence: high (four plus: ⊕⊕⊕⊕), moderate (three plus: ⊕⊕⊕◯), low (two plus: ⊕⊕◯◯), and very low (one plus: ⊕◯◯◯).

Overview

We used the GRADE framework to assess the certainty of evidence at the outcome level for each core outcome included in the meta-analysis, including FMA-UE, ARAT, FMA-UE proximal, FMA-UE distal, and MAS-finger. Since all included studies were randomized controlled trials (RCTs), the initial certainty of evidence for each outcome was rated as "high," followed by downgrading assessments across five domains: risk of bias, inconsistency, indirectness, imprecision, and publication bias. For each outcome, we summarized the pooled effect size (MD) and corresponding 95% confidence interval, number of studies and sample size, heterogeneity statistic (I^2), and qualitative characteristics (e.g., consistency of outcome measurements and follow-up time points). These data informed the domain-specific judgments and final certainty ratings (high, moderate, low, or very low).

Decision rules

Risk of bias (study limitations): Evidence was downgraded by one level when most included studies were judged as having "high risk" or "some concerns" in key methodological domains (e.g., inadequate allocation concealment or lack of blinding). In this review, most studies had limitations in allocation concealment and blinding, although the RCT design itself provided a high initial level of evidence.

Inconsistency (heterogeneity): Evidence was downgraded by one level when $I^2 \geqslant 50\%$ with minimal overlap of confidence intervals and inconsistent directions of effect; downgraded by two levels when $I^2 \geqslant 75\%$ with clearly conflicting effects; and not downgraded when $I^2 < 50\%$ with substantial overlap of confidence intervals and consistent effect directions.

Indirectness (PICO differences): Evidence was downgraded by one level when there were important differences in population, intervention, comparator, or outcomes between the included studies and the review question. For outcomes measured using ordinal scales (e.g., MAS), downgrading for indirectness was also considered when the scale properties were not fully compatible with continuous-data analysis methods.

Imprecision (random error): Evidence was downgraded by one level when the 95% confidence interval crossed the null effect and simultaneously included the minimal clinically important difference (MCID) range, or when the total sample size did not meet the optimal information size (OIS) criterion (total sample size <400 participants for continuous outcomes). Evidence was

downgraded by two levels for very serious imprecision when both conditions were met simultaneously.

Publication bias: Funnel plot analysis was not performed because fewer than 10 studies were included for each outcome. Unless there was other clear evidence suggesting small-study effects or selective reporting, the certainty of evidence was not downgraded for publication bias.

	Experimental			Control				Mean Difference
**Study or Subgroup**	**Mean**	**SD**	**Total**	**Mean**	**SD**	**Total**	**Weight**	**IV, Random, 95% CI**
Ang K K 2014	40.2	17.46	6	32.8	14.44	8	4.9%	7.40 [−9.78, 24.58]
Cantillo-Negrete J 2025	31.33	17.2	10	32.6	25.54	9	3.7%	−1.27 [−21.07, 18.53]
Cheng N 2020	28	10.74	5	35.6	9.42	5	9.3%	−7.60 [−20.12, 4.92]
Frolov A A 2017	30	25.12	55	26.67	31.24	19	6.1%	3.33 [−12.21, 18.87]
Guo N 2022	28.6	14.03	10	21.9	7.62	10	14.9%	6.70 [−3.20, 16.60]
Ji X 2025	41.25	19.75	20	33.33	16.82	19	11.1%	7.92 [−3.57, 19.41]
Liu M Y 2023	33.58	8.47	19	27.22	8.31	18	50.0%	6.36 [0.95, 11.77]
**Total (95% CI)**			**125**			**88**	**100.0%**	**4.87 [1.04, 8.69]**

Heterogeneity: $Tau^2 = 0.00$; $Chi^2 = 4.99$, df = 6 (P = 0.54); $I^2 = 0\%$
Test for overall effect: Z = 2.49 (P = 0.01)

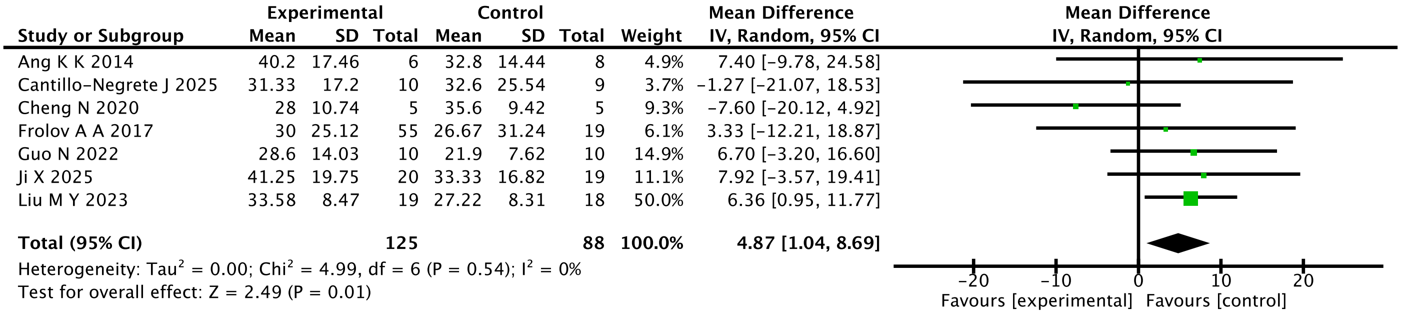

Supplementary Figure S1. Forest plot of FMA-UE: BCI-robot vs. robot.

	Experimental			Control				Mean Difference
**Study or Subgroup**	**Mean**	**SD**	**Total**	**Mean**	**SD**	**Total**	**Weight**	**IV, Random, 95% CI**
Ang K K 2014	40.2	17.46	6	28.3	16.93	7	2.7%	11.90 [−6.87, 30.67]
Cantillo-Negrete J 2021	23.1	16.1	10	21.9	15.5	10	4.9%	1.20 [−12.65, 15.05]
Fu J 2023	31.25	22.77	30	31.67	22.5	31	7.2%	−0.42 [−11.78, 10.94]
Guo N 2022	28.6	14.03	10	15.2	9.48	10	8.4%	13.40 [2.91, 23.89]
Wu Q 2020	35.36	4.26	14	28.07	4.83	11	67.4%	7.29 [3.67, 10.91]
Zanona A F 2023	42.6	18.8	23	40.9	14.7	21	9.4%	1.70 [−8.23, 11.63]
**Total (95% CI)**			**93**			**90**	**100.0%**	**6.55 [3.49, 9.61]**

Heterogeneity: $Tau^2 = 0.20$; $Chi^2 = 5.04$, df = 5 (P = 0.41); $I^2 = 1\%$
Test for overall effect: Z = 4.19 (P < 0.0001)

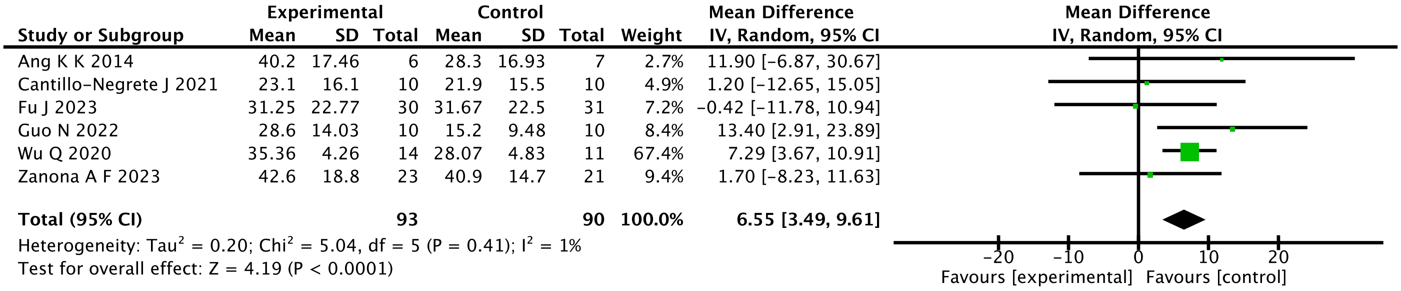

Supplementary Figure S2. Forest plot of FMA-UE: BCI-robot vs. conventional rehabilitation.

	Experimental			Control				Mean Difference
**Study or Subgroup**	**Mean**	**SD**	**Total**	**Mean**	**SD**	**Total**	**Weight**	**IV, Random, 95% CI**
Cantillo-Negrete J 2025	21	24.94	10	19.43	33.5	9	4.8%	1.57 [−25.22, 28.36]
Cheng N 2020	5.4	4.8	5	9	8.85	5	44.4%	−3.60 [−12.42, 5.22]
Frolov A A 2017	16.67	31.97	55	12.33	24.83	19	17.6%	4.34 [−9.66, 18.34]
Ji X 2025	20.58	19.75	20	12.67	12.01	19	33.2%	7.91 [−2.29, 18.11]
**Total (95% CI)**			**90**			**52**	**100.0%**	**1.87 [−4.01, 7.75]**

Heterogeneity: $Tau^2 = 0.00$; $Chi^2 = 2.94$, df = 3 (P = 0.40); $I^2 = 0\%$
Test for overall effect: Z = 0.62 (P = 0.53)

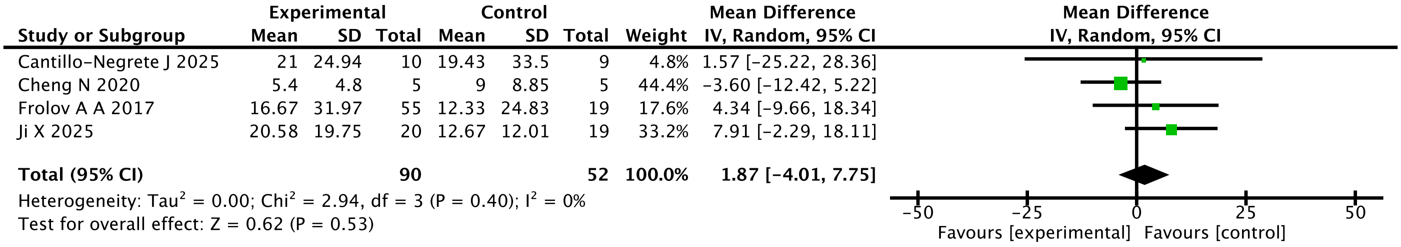

Supplementary Figure S3. Forest plot of ARAT: BCI-robot vs. robot.

	Experimental			Control				Mean Difference
**Study or Subgroup**	**Mean**	**SD**	**Total**	**Mean**	**SD**	**Total**	**Weight**	**IV, Random, 95% CI**
Cantillo-Negrete J 2021	8.4	10.1	10	8.7	11.3	10	51.1%	−0.30 [−9.69, 9.09]
Wu Q 2020	28.07	4.83	14	10.33	17.78	11	48.9%	17.74 [6.93, 28.55]
**Total (95% CI)**			**24**			**21**	**100.0%**	**8.51 [−9.16, 26.19]**

Heterogeneity: $Tau^2 = 136.03$; $Chi^2 = 6.10$, df = 1 (P = 0.01); $I^2 = 84\%$
Test for overall effect: Z = 0.94 (P = 0.35)

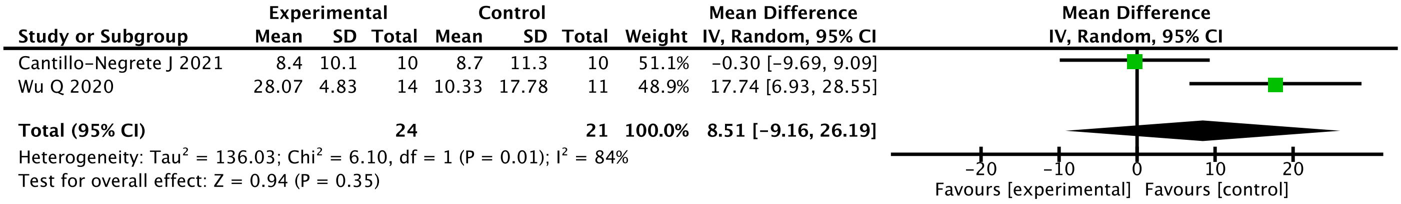

Supplementary Figure S4. Forest plot of ARAT: BCI-robot vs. conventional rehabilitation.

Study or Subgroup	Experimental Mean	Experimental SD	Experimental Total	Control Mean	Control SD	Control Total	Weight	Mean Difference IV, Random, 95% CI
Ang K K 2014	28	9.15	6	23.9	9.35	8	19.3%	4.10 [−5.68, 13.88]
Frolov A A 2017	23	14.46	55	18	13.62	19	35.4%	5.00 [−2.22, 12.22]
Guo N 2022	20.7	9.46	10	16.55	4.07	10	45.3%	4.15 [−2.23, 10.53]
**Total (95% CI)**			**71**			**37**	**100.0%**	**4.44 [0.15, 8.74]**

Heterogeneity: $Tau^2 = 0.00$; $Chi^2 = 0.04$, df = 2 (P = 0.98); $I^2 = 0\%$
Test for overall effect: Z = 2.03 (P = 0.04)

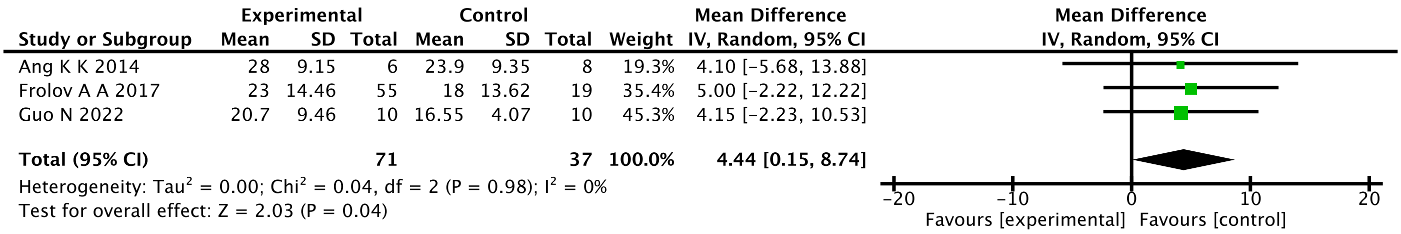

Supplementary Figure S5. Forest plot of FMA-UE proximal score: BCI-robot vs. robot.

Study or Subgroup	Experimental Mean	Experimental SD	Experimental Total	Control Mean	Control SD	Control Total	Weight	Mean Difference IV, Random, 95% CI
Ang K K 2014	28	9.15	6	21.1	11.98	7	27.2%	6.90 [−4.60, 18.40]
Guo N 2022	20.7	9.46	10	12.4	6.24	10	72.8%	8.30 [1.28, 15.32]
**Total (95% CI)**			**16**			**17**	**100.0%**	**7.92 [1.92, 13.91]**

Heterogeneity: $Tau^2 = 0.00$; $Chi^2 = 0.04$, df = 1 (P = 0.84); $I^2 = 0\%$
Test for overall effect: Z = 2.59 (P = 0.010)

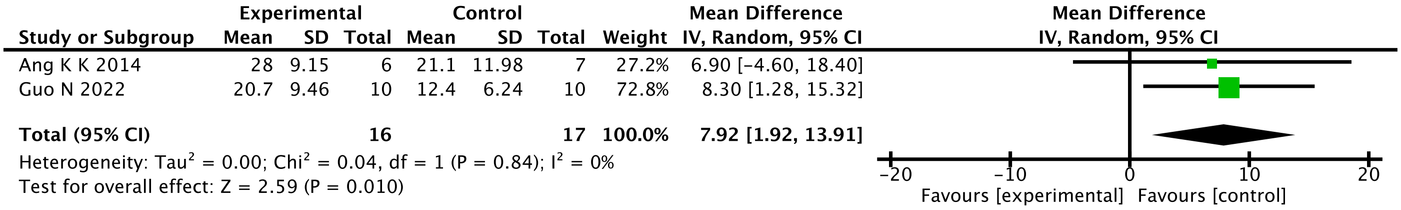

Supplementary Figure S6. Forest plot of FMA-UE proximal score: BCI-robot vs. conventional rehabilitation.

Study or Subgroup	Experimental Mean	Experimental SD	Experimental Total	Control Mean	Control SD	Control Total	Weight	Mean Difference IV, Random, 95% CI
Ang K K 2014	12.1	10.54	6	8.9	6.72	8	0.8%	3.20 [−6.43, 12.83]
Frolov A A 2017	9	12.18	55	6.67	12.01	19	1.9%	2.33 [−3.96, 8.62]
Guo N 2022	7.9	6.06	10	5.35	4	10	3.8%	2.55 [−1.95, 7.05]
Liu M Y 2023	5.25	1.4	19	5.25	1.41	18	93.4%	0.00 [−0.91, 0.91]
**Total (95% CI)**			**90**			**55**	**100.0%**	**0.17 [−0.71, 1.04]**

Heterogeneity: $Tau^2 = 0.00$; $Chi^2 = 2.04$, df = 3 (P = 0.56); $I^2 = 0\%$
Test for overall effect: Z = 0.38 (P = 0.71)

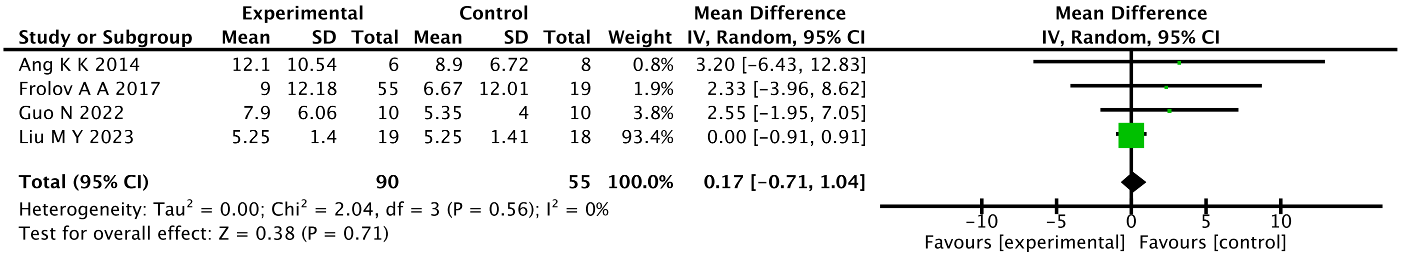

Supplementary Figure S7. Forest plot of FMA-UE distal score: BCI-robot vs. robot.

Study or Subgroup	Experimental Mean	Experimental SD	Experimental Total	Control Mean	Control SD	Control Total	Weight	Mean Difference IV, Random, 95% CI
Ang K K 2014	12.1	10.54	6	7.2	5.88	7	17.8%	4.90 [−4.59, 14.39]
Guo N 2022	7.9	6.06	10	2.8	3.77	10	82.2%	5.10 [0.68, 9.52]
**Total (95% CI)**			**16**			**17**	**100.0%**	**5.06 [1.05, 9.07]**

Heterogeneity: $Tau^2 = 0.00$; $Chi^2 = 0.00$, df = 1 (P = 0.97); $I^2 = 0\%$
Test for overall effect: Z = 2.48 (P = 0.01)

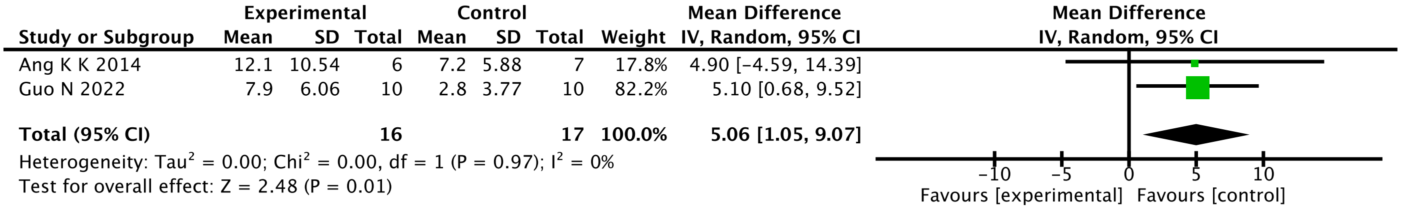

Supplementary Figure S8. Forest plot of FMA-UE distal score: BCI-robot vs. conventional rehabilitation.

Study or Subgroup	Experimental Mean	Experimental SD	Experimental Total	Control Mean	Control SD	Control Total	Weight	Mean Difference IV, Random, 95% CI
Guo N 2022	0.45	0.6	10	0.65	0.64	10	18.4%	−0.20 [−0.74, 0.34]
Liu M Y 2023	1.17	0.4	19	1.67	0.4	18	81.6%	−0.50 [−0.76, −0.24]
**Total (95% CI)**			**29**			**28**	**100.0%**	**−0.44 [−0.68, −0.21]**

Heterogeneity: $Tau^2 = 0.00$; $Chi^2 = 0.95$, df = 1 (P = 0.33); $I^2 = 0\%$
Test for overall effect: Z = 3.74 (P = 0.0002)

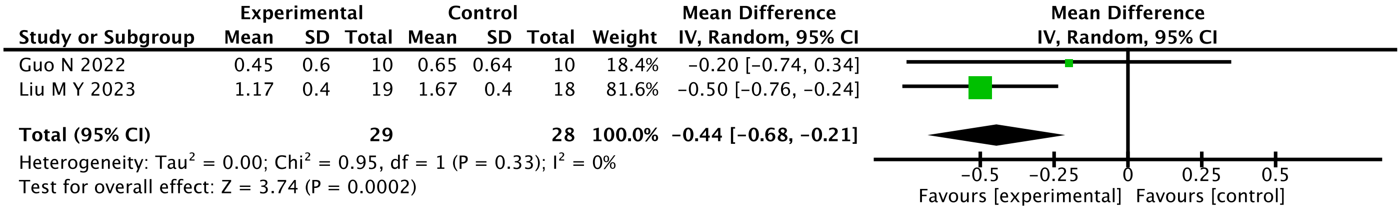

Supplementary Figure S9. Forest plot of MAS finger flexor score: BCI-robot vs. robot.

Study or Subgroup	Experimental Mean	Experimental SD	Experimental Total	Control Mean	Control SD	Control Total	Weight	Mean Difference IV, Random, 95% CI
**7.1.1 subacute stroke**								
Frolov A A 2017	25.67	25.94	25	20	16.62	9	6.7%	5.67 [−9.21, 20.55]
Ji X 2025	41.25	19.75	20	33.33	16.82	19	11.1%	7.92 [−3.57, 19.41]
Liu M Y 2023	33.58	8.47	19	27.22	8.31	18	50.4%	6.36 [0.95, 11.77]
**Subtotal (95% CI)**			**64**			**46**	**68.2%**	**6.55 [1.90, 11.20]**
Heterogeneity: $Tau^2 = 0.00$; $Chi^2 = 0.07$, df = 2 (P = 0.96); $I^2 = 0\%$								
Test for overall effect: Z = 2.76 (P = 0.006)								
**7.1.2 chronic stroke**								
Ang K K 2014	40.2	17.46	6	32.8	14.44	8	5.0%	7.40 [−9.78, 24.58]
Cheng N 2020	28	10.74	5	35.6	9.42	5	9.4%	−7.60 [−20.12, 4.92]
Frolov A A 2017	36.67	26.47	30	28.17	36.98	10	2.4%	8.50 [−16.30, 33.30]
Guo N 2022	28.6	14.03	10	21.9	7.62	10	15.0%	6.70 [−3.20, 16.60]
**Subtotal (95% CI)**			**51**			**33**	**31.8%**	**2.67 [−5.25, 10.60]**
Heterogeneity: $Tau^2 = 13.24$; $Chi^2 = 3.72$, df = 3 (P = 0.29); $I^2 = 19\%$								
Test for overall effect: Z = 0.66 (P = 0.51)								
**Total (95% CI)**			**115**			**79**	**100.0%**	**5.33 [1.49, 9.17]**
Heterogeneity: $Tau^2 = 0.00$; $Chi^2 = 4.62$, df = 6 (P = 0.59); $I^2 = 0\%$								
Test for overall effect: Z = 2.72 (P = 0.006)								
Test for subgroup differences: $Chi^2 = 0.68$, df = 1 (P = 0.41), $I^2 = 0\%$								

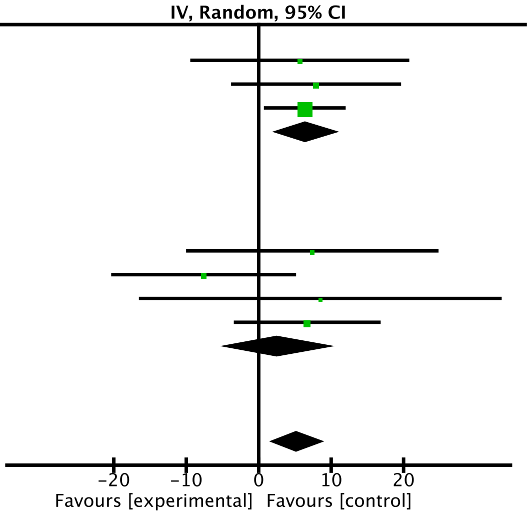

Supplementary Figure S10. Forest plot of subgroup analysis by stroke stage for FMA-UE: BCI-robot vs. robot.

Study or Subgroup	Experimental Mean	Experimental SD	Experimental Total	Control Mean	Control SD	Control Total	Weight	Mean Difference IV, Random, 95% CI
**7.2.1 subacute stroke**								
Wu Q 2020	35.36	4.26	14	28.07	4.83	11	77.5%	7.29 [3.67, 10.91]
Zanona A F 2023	42.6	18.8	23	40.9	14.7	21	10.3%	1.70 [−8.23, 11.63]
**Subtotal (95% CI)**			**37**			**32**	**87.9%**	**6.48 [2.64, 10.33]**
Heterogeneity: $Tau^2 = 1.09$; $Chi^2 = 1.07$, df = 1 (P = 0.30); $I^2 = 7\%$								
Test for overall effect: Z = 3.30 (P = 0.0010)								
**7.2.2 chronic stroke**								
Ang K K 2014	40.2	17.46	6	28.3	16.93	7	2.9%	11.90 [−6.87, 30.67]
Guo N 2022	28.6	14.03	10	15.2	9.48	10	9.2%	13.40 [2.91, 23.89]
**Subtotal (95% CI)**			**16**			**17**	**12.1%**	**13.04 [3.88, 22.20]**
Heterogeneity: $Tau^2 = 0.00$; $Chi^2 = 0.02$, df = 1 (P = 0.89); $I^2 = 0\%$								
Test for overall effect: Z = 2.79 (P = 0.005)								
**Total (95% CI)**			**53**			**49**	**100.0%**	**7.41 [4.22, 10.60]**
Heterogeneity: $Tau^2 = 0.00$; $Chi^2 = 2.75$, df = 3 (P = 0.43); $I^2 = 0\%$								
Test for overall effect: Z = 4.55 (P < 0.00001)								
Test for subgroup differences: $Chi^2 = 1.67$, df = 1 (P = 0.20), $I^2 = 40.3\%$								

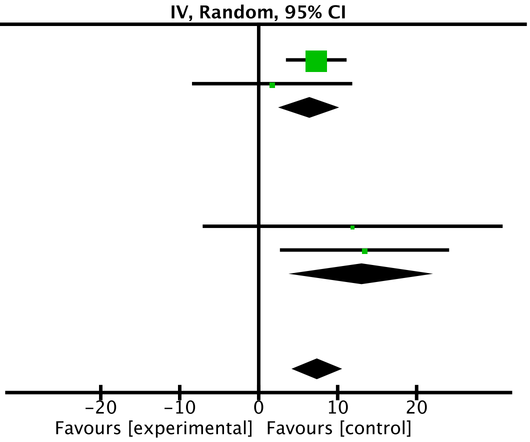

Supplementary Figure S11. Forest plot of subgroup analysis by stroke stage for FMA-UE: BCI-robot vs. conventional rehabilitation.

Study or Subgroup	Experimental Mean	Experimental SD	Experimental Total	Control Mean	Control SD	Control Total	Weight	Mean Difference IV, Random, 95% CI
**7.3.1 subacute stroke**								
Frolov A A 2017	8	16.51	25	12	20.99	9	18.4%	−4.00 [−19.16, 11.16]
Ji X 2025	20.58	19.75	20	12.67	12.01	19	32.9%	7.91 [−2.29, 18.11]
**Subtotal (95% CI)**			**45**			**28**	**51.3%**	**3.33 [−8.03, 14.69]**
Heterogeneity: $Tau^2 = 27.45$; $Chi^2 = 1.63$, df = 1 (P = 0.20); $I^2 = 39\%$								
Test for overall effect: Z = 0.57 (P = 0.57)								
**7.3.2 chronic stroke**								
Cheng N 2020	5.4	4.8	5	9	8.85	5	39.3%	−3.60 [−12.42, 5.22]
Frolov A A 2017	25	32.69	30	13.33	30.96	10	9.4%	11.67 [−10.80, 34.14]
**Subtotal (95% CI)**			**35**			**15**	**48.7%**	**0.39 [−12.76, 13.55]**
Heterogeneity: $Tau^2 = 40.71$; $Chi^2 = 1.54$, df = 1 (P = 0.22); $I^2 = 35\%$								
Test for overall effect: Z = 0.06 (P = 0.95)								
**Total (95% CI)**			**80**			**43**	**100.0%**	**1.55 [−5.73, 8.82]**
Heterogeneity: $Tau^2 = 14.81$; $Chi^2 = 4.08$, df = 3 (P = 0.25); $I^2 = 27\%$								
Test for overall effect: Z = 0.42 (P = 0.68)								
Test for subgroup differences: $Chi^2 = 0.11$, df = 1 (P = 0.74), $I^2 = 0\%$								

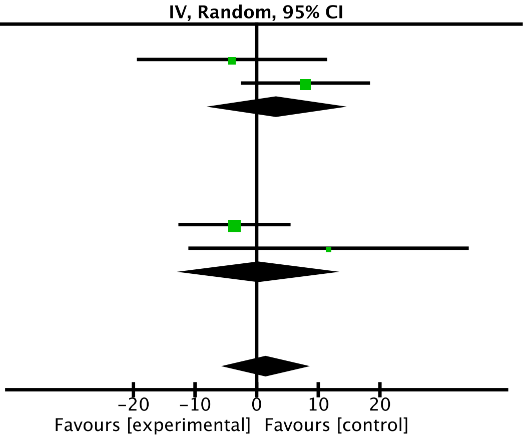

Supplementary Figure S12. Forest plot of subgroup analysis by stroke stage for ARAT: BCI-robot vs. robot.

Study or Subgroup	Experimental Mean	Experimental SD	Experimental Total	Control Mean	Control SD	Control Total	Weight	Mean Difference IV, Random, 95% CI
**8.1.1 Exoskeleton**								
Cantillo-Negrete J 2025	31.33	17.2	10	32.6	25.54	9	7.4%	-1.27 [-21.07, 18.53]
Frolov A A 2017	30	25.12	55	26.67	31.24	19	12.1%	3.33 [-12.21, 18.87]
**Subtotal (95% CI)**			**65**			**28**	**19.5%**	**1.58 [-10.65, 13.80]**
Heterogeneity: Tau^2 = 0.00; Chi^2 = 0.13, df = 1 (P = 0.72); I^2 = 0%								
Test for overall effect: Z = 0.25 (P = 0.80)								
**8.1.2 Soft Robotic Glove**								
Cheng N 2020	28	10.74	5	35.6	9.42	5	18.6%	-7.60 [-20.12, 4.92]
Guo N 2022	28.6	14.03	10	21.9	7.62	10	29.8%	6.70 [-3.20, 16.60]
Ji X 2025	41.25	19.75	20	33.33	16.82	19	22.1%	7.92 [-3.57, 19.41]
**Subtotal (95% CI)**			**35**			**34**	**70.6%**	**2.84 [-6.33, 12.01]**
Heterogeneity: Tau^2 = 32.73; Chi^2 = 3.98, df = 2 (P = 0.14); I^2 = 50%								
Test for overall effect: Z = 0.61 (P = 0.54)								
**8.1.3 End Effector**								
Ang K K 2014	40.2	17.46	6	32.8	14.44	8	9.9%	7.40 [-9.78, 24.58]
**Subtotal (95% CI)**			**6**			**8**	**9.9%**	**7.40 [-9.78, 24.58]**
Heterogeneity: Not applicable								
Test for overall effect: Z = 0.84 (P = 0.40)								
**Total (95% CI)**			**106**			**70**	**100.0%**	**3.37 [-2.03, 8.78]**
Heterogeneity: Tau^2 = 0.00; Chi^2 = 4.41, df = 5 (P = 0.49); I^2 = 0%								
Test for overall effect: Z = 1.22 (P = 0.22)								
Test for subgroup differences: Chi^2 = 0.30, df = 2 (P = 0.86), I^2 = 0%								

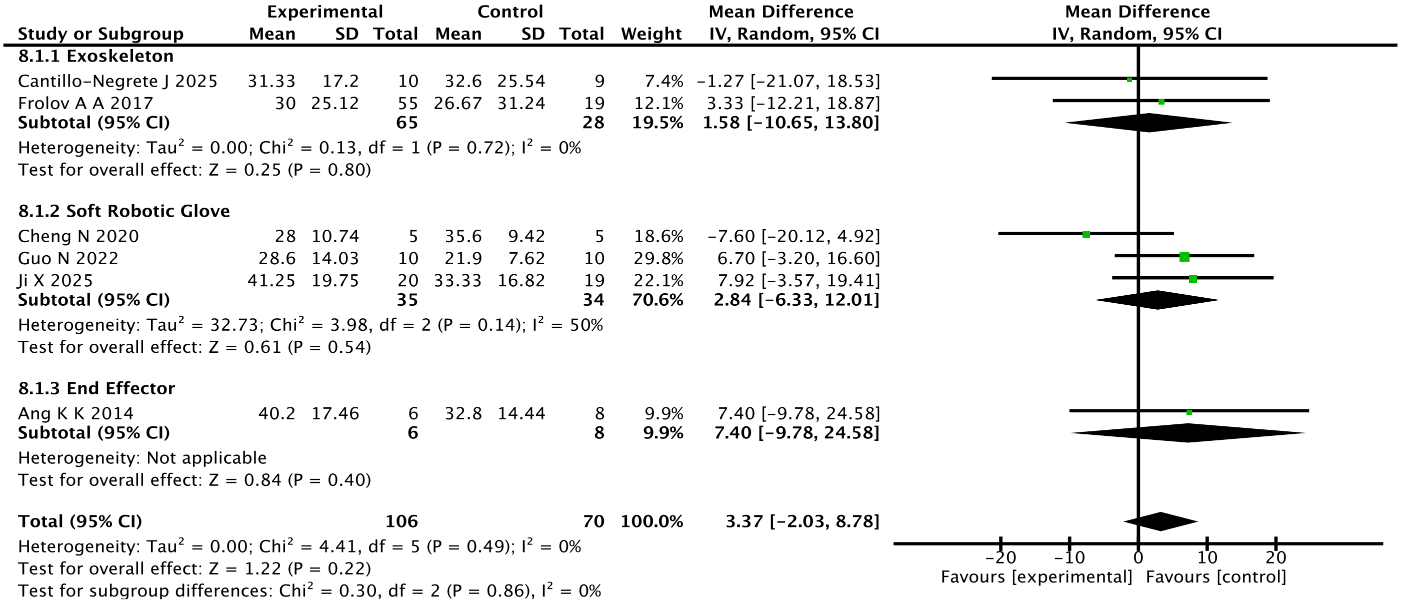

Supplementary Figure S13. Forest plot of subgroup analysis by robot type for FMA-UE: BCI-robot vs. robot.

Study or Subgroup	Experimental Mean	Experimental SD	Experimental Total	Control Mean	Control SD	Control Total	Weight	Mean Difference IV, Random, 95% CI
**8.2.1 Exoskeleton**								
Cantillo-Negrete J 2021	23.1	16.1	10	21.9	15.5	10	4.9%	1.20 [-12.65, 15.05]
Fu J 2023	31.25	22.77	30	31.67	22.5	31	7.2%	-0.42 [-11.78, 10.94]
Wu Q 2020	35.36	4.26	14	28.07	4.83	11	67.4%	7.29 [3.67, 10.91]
Zanona A F 2023	42.6	18.8	23	40.9	14.7	21	9.4%	1.70 [-8.23, 11.63]
**Subtotal (95% CI)**			**77**			**73**	**88.9%**	**5.80 [2.62, 8.97]**
Heterogeneity: Tau^2 = 0.00; Chi^2 = 2.88, df = 3 (P = 0.41); I^2 = 0%								
Test for overall effect: Z = 3.58 (P = 0.0003)								
**8.2.2 Soft Robotic Glove**								
Guo N 2022	28.6	14.03	10	15.2	9.48	10	8.4%	13.40 [2.91, 23.89]
**Subtotal (95% CI)**			**10**			**10**	**8.4%**	**13.40 [2.91, 23.89]**
Heterogeneity: Not applicable								
Test for overall effect: Z = 2.50 (P = 0.01)								
**8.2.3 End Effector**								
Ang K K 2014	40.2	17.46	6	28.3	16.93	7	2.7%	11.90 [-6.87, 30.67]
**Subtotal (95% CI)**			**6**			**7**	**2.7%**	**11.90 [-6.87, 30.67]**
Heterogeneity: Not applicable								
Test for overall effect: Z = 1.24 (P = 0.21)								
**Total (95% CI)**			**93**			**90**	**100.0%**	**6.55 [3.49, 9.61]**
Heterogeneity: Tau^2 = 0.20; Chi^2 = 5.04, df = 5 (P = 0.41); I^2 = 1%								
Test for overall effect: Z = 4.19 (P < 0.0001)								
Test for subgroup differences: Chi^2 = 2.16, df = 2 (P = 0.34), I^2 = 7.6%								

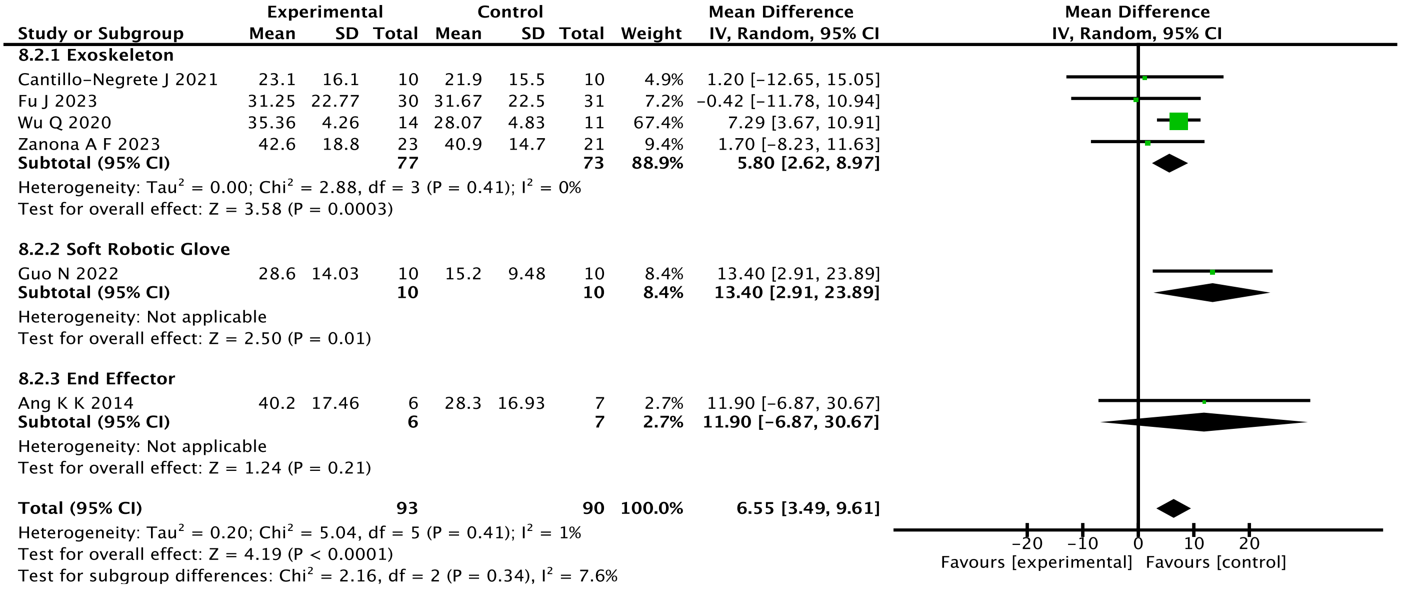

Supplementary Figure S14. Forest plot of subgroup analysis by robot type for FMA-UE: BCI-robot vs. conventional rehabilitation.

Study or Subgroup	Experimental Mean	Experimental SD	Experimental Total	Control Mean	Control SD	Control Total	Weight	Mean Difference IV, Random, 95% CI
**8.3.1 Exoskeleton**								
Cantillo-Negrete J 2025	21	24.94	10	19.43	33.5	9	4.8%	1.57 [-25.22, 28.36]
Frolov A A 2017	16.67	31.97	55	12.33	24.83	19	17.6%	4.34 [-9.66, 18.34]
**Subtotal (95% CI)**			**65**			**28**	**22.4%**	**3.75 [-8.66, 16.16]**
Heterogeneity: Tau^2 = 0.00; Chi^2 = 0.03, df = 1 (P = 0.86); I^2 = 0%								
Test for overall effect: Z = 0.59 (P = 0.55)								
**8.3.2 Soft Robotic Glove**								
Cheng N 2020	5.4	4.8	5	9	8.85	5	44.4%	-3.60 [-12.42, 5.22]
Ji X 2025	20.58	19.75	20	12.67	12.01	19	33.2%	7.91 [-2.29, 18.11]
**Subtotal (95% CI)**			**25**			**24**	**77.6%**	**1.86 [-9.41, 13.12]**
Heterogeneity: Tau^2 = 42.56; Chi^2 = 2.80, df = 1 (P = 0.09); I^2 = 64%								
Test for overall effect: Z = 0.32 (P = 0.75)								
**Total (95% CI)**			**90**			**52**	**100.0%**	**1.87 [-4.01, 7.75]**
Heterogeneity: Tau^2 = 0.00; Chi^2 = 2.94, df = 3 (P = 0.40); I^2 = 0%								
Test for overall effect: Z = 0.62 (P = 0.53)								
Test for subgroup differences: Chi^2 = 0.05, df = 1 (P = 0.83), I^2 = 0%								

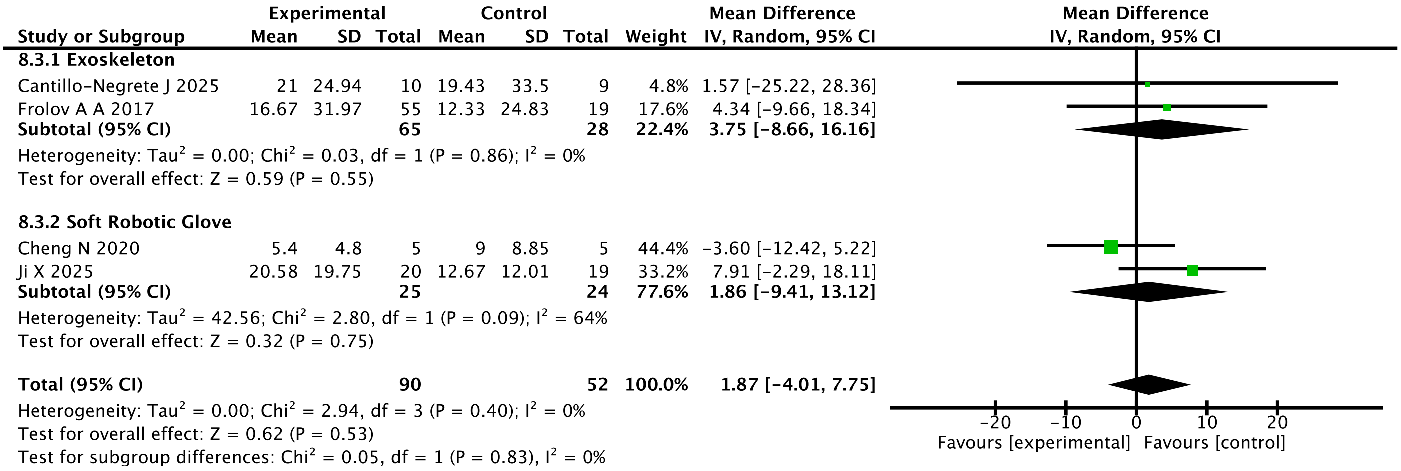

Supplementary Figure S15. Forest plot of subgroup analysis by robot type for ARAT: BCI-robot vs. robot.

Study or Subgroup	Experimental Mean	Experimental SD	Experimental Total	Control Mean	Control SD	Control Total	Weight	Mean Difference IV, Random, 95% CI
**9.1.1 MI-BCI**								
Ang K K 2014	40.2	17.46	6	32.8	14.44	8	4.9%	7.40 [-9.78, 24.58]
Cantillo-Negrete J 2025	31.33	17.2	10	32.6	25.54	9	3.7%	-1.27 [-21.07, 18.53]
Cheng N 2020	28	10.74	5	35.6	9.42	5	9.3%	-7.60 [-20.12, 4.92]
Frolov A A 2017	30	25.12	55	26.67	31.24	19	6.1%	3.33 [-12.21, 18.87]
Ji X 2025	41.25	19.75	20	33.33	16.82	19	11.1%	7.92 [-3.57, 19.41]
Liu M Y 2023	33.58	8.47	19	27.22	8.31	18	50.0%	6.36 [0.95, 11.77]
**Subtotal (95% CI)**			**115**			**78**	**85.1%**	**4.54 [0.40, 8.69]**
Heterogeneity: $Tau^2 = 0.00$; $Chi^2 = 4.84$, df = 5 (P = 0.44); $I^2 = 0\%$								
Test for overall effect: Z = 2.15 (P = 0.03)								
**9.1.2 SSVEP-BCI**								
Guo N 2022	28.6	14.03	10	21.9	7.62	10	14.9%	6.70 [-3.20, 16.60]
**Subtotal (95% CI)**			**10**			**10**	**14.9%**	**6.70 [-3.20, 16.60]**
Heterogeneity: Not applicable								
Test for overall effect: Z = 1.33 (P = 0.18)								
**Total (95% CI)**			**125**			**88**	**100.0%**	**4.87 [1.04, 8.69]**
Heterogeneity: $Tau^2 = 0.00$; $Chi^2 = 4.99$, df = 6 (P = 0.54); $I^2 = 0\%$								
Test for overall effect: Z = 2.49 (P = 0.01)								
Test for subgroup differences: $Chi^2 = 0.16$, df = 1 (P = 0.69), $I^2 = 0\%$								

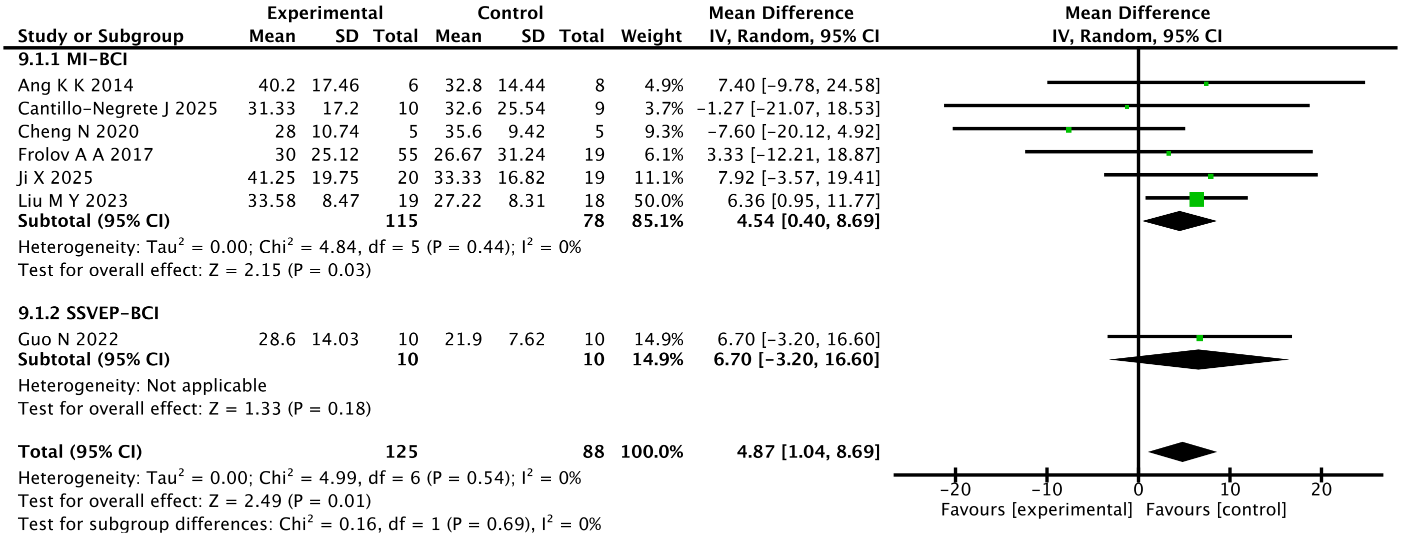

Supplementary Figure S16. Forest plot of subgroup analysis by BCI paradigm for FMA-UE: BCI-robot vs. robot.

Study or Subgroup	Experimental Mean	Experimental SD	Experimental Total	Control Mean	Control SD	Control Total	Weight	Mean Difference IV, Random, 95% CI
**9.2.1 MI-BCI**								
Ang K K 2014	40.2	17.46	6	28.3	16.93	7	2.7%	11.90 [-6.87, 30.67]
Cantillo-Negrete J 2021	23.1	16.1	10	21.9	15.5	10	4.9%	1.20 [-12.65, 15.05]
Wu Q 2020	35.36	4.26	14	28.07	4.83	11	67.4%	7.29 [3.67, 10.91]
Zanona A F 2023	42.6	18.8	23	40.9	14.7	21	9.4%	1.70 [-8.23, 11.63]
**Subtotal (95% CI)**			**53**			**49**	**84.3%**	**6.49 [3.24, 9.75]**
Heterogeneity: $Tau^2 = 0.00$; $Chi^2 = 1.96$, df = 3 (P = 0.58); $I^2 = 0\%$								
Test for overall effect: Z = 3.91 (P < 0.0001)								
**9.2.2 SSVEP-BCI**								
Guo N 2022	28.6	14.03	10	15.2	9.48	10	8.4%	13.40 [2.91, 23.89]
**Subtotal (95% CI)**			**10**			**10**	**8.4%**	**13.40 [2.91, 23.89]**
Heterogeneity: Not applicable								
Test for overall effect: Z = 2.50 (P = 0.01)								
**9.2.3 MA-BCI**								
Fu J 2023	31.25	22.77	30	31.67	22.5	31	7.2%	-0.42 [-11.78, 10.94]
**Subtotal (95% CI)**			**30**			**31**	**7.2%**	**-0.42 [-11.78, 10.94]**
Heterogeneity: Not applicable								
Test for overall effect: Z = 0.07 (P = 0.94)								
**Total (95% CI)**			**93**			**90**	**100.0%**	**6.55 [3.49, 9.61]**
Heterogeneity: $Tau^2 = 0.20$; $Chi^2 = 5.04$, df = 5 (P = 0.41); $I^2 = 1\%$								
Test for overall effect: Z = 4.19 (P < 0.0001)								
Test for subgroup differences: $Chi^2 = 3.08$, df = 2 (P = 0.21), $I^2 = 35.1\%$								

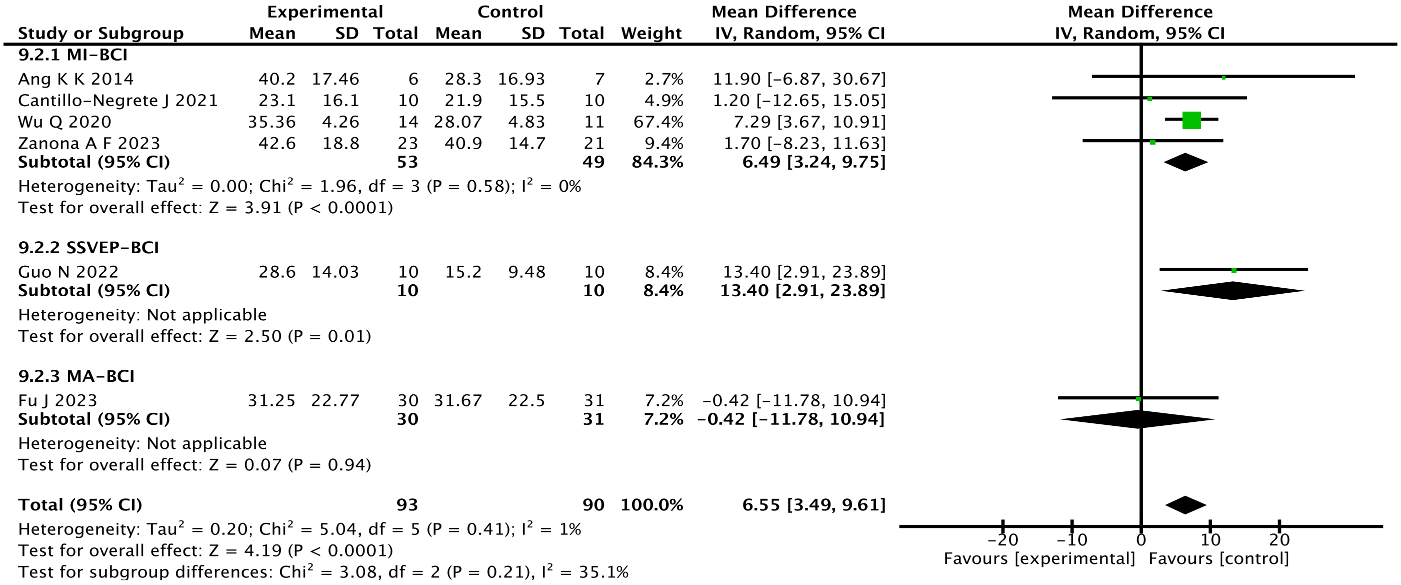

Supplementary Figure S17. Forest plot of subgroup analysis by BCI paradigm for FMA-UE: BCI-robot vs. conventional rehabilitation.

Study or Subgroup	Experimental Mean	Experimental SD	Experimental Total	Control Mean	Control SD	Control Total	Weight	Mean Difference IV, Random, 95% CI
**10.1.1 Moderate to severe**								
Ang K K 2014	40.2	17.46	6	32.8	14.44	8	4.9%	7.40 [-9.78, 24.58]
Cantillo-Negrete J 2025	31.33	17.2	10	32.6	25.54	9	3.7%	-1.27 [-21.07, 18.53]
Cheng N 2020	28	10.74	5	35.6	9.42	5	9.3%	-7.60 [-20.12, 4.92]
Guo N 2022	28.6	14.03	10	21.9	7.62	10	14.9%	6.70 [-3.20, 16.60]
Ji X 2025	41.25	19.75	20	33.33	16.82	19	11.1%	7.92 [-3.57, 19.41]
Liu M Y 2023	33.58	8.47	19	27.22	8.31	18	50.0%	6.36 [0.95, 11.77]
**Subtotal (95% CI)**			**70**			**69**	**93.9%**	**4.96 [1.02, 8.91]**
Heterogeneity: $Tau^2 = 0.00$; $Chi^2 = 4.95$, df = 5 (P = 0.42); $I^2 = 0\%$								
Test for overall effect: Z = 2.47 (P = 0.01)								
**10.1.2 Severe**								
Frolov A A 2017	30	25.12	55	26.67	31.24	19	6.1%	3.33 [-12.21, 18.87]
**Subtotal (95% CI)**			**55**			**19**	**6.1%**	**3.33 [-12.21, 18.87]**
Heterogeneity: Not applicable								
Test for overall effect: Z = 0.42 (P = 0.67)								
**Total (95% CI)**			**125**			**88**	**100.0%**	**4.87 [1.04, 8.69]**
Heterogeneity: $Tau^2 = 0.00$; $Chi^2 = 4.99$, df = 6 (P = 0.54); $I^2 = 0\%$								
Test for overall effect: Z = 2.49 (P = 0.01)								
Test for subgroup differences: $Chi^2 = 0.04$, df = 1 (P = 0.84), $I^2 = 0\%$								

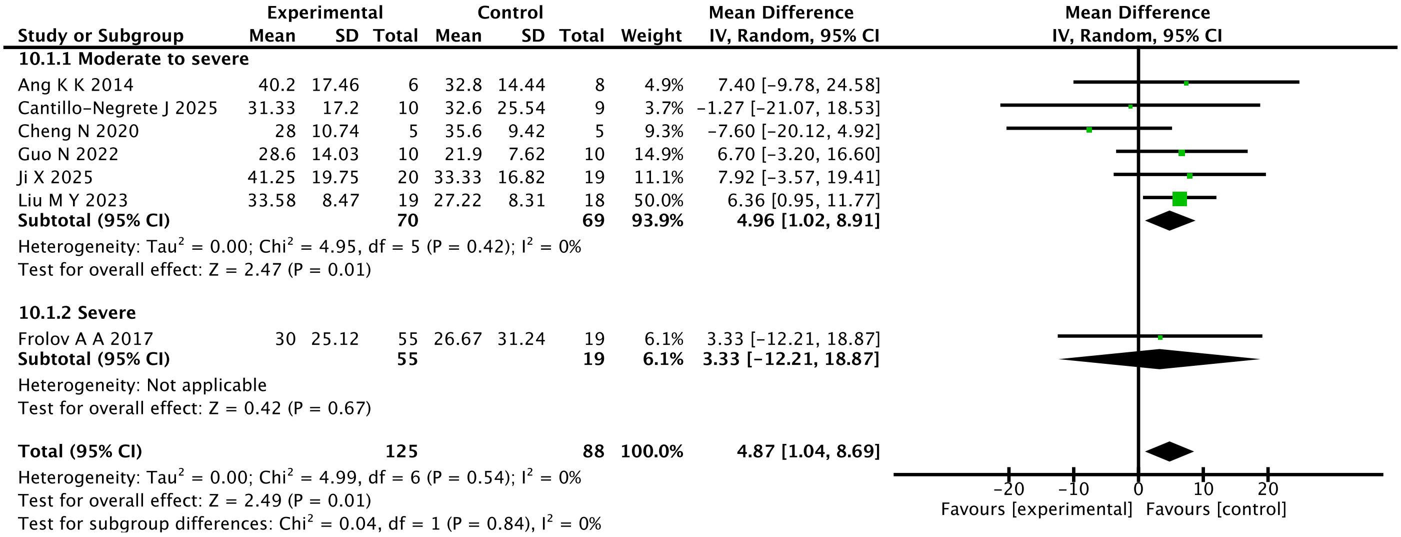

Supplementary Figure S18. Forest plot of subgroup analysis by upper limb impairment severity for FMA-UE: BCI-robot vs. robot.

Study or Subgroup	Experimental Mean	Experimental SD	Experimental Total	Control Mean	Control SD	Control Total	Weight	Mean Difference IV, Random, 95% CI
**10.2.1 Moderate to severe**								
Ang K K 2014	40.2	17.46	6	28.3	16.93	7	2.7%	11.90 [-6.87, 30.67]
Guo N 2022	28.6	14.03	10	15.2	9.48	10	8.4%	13.40 [2.91, 23.89]
Wu Q 2020	35.36	4.26	14	28.07	4.83	11	67.4%	7.29 [3.67, 10.91]
Zanona A F 2023	42.6	18.8	23	40.9	14.7	21	9.4%	1.70 [-8.23, 11.63]
**Subtotal (95% CI)**			**53**			**49**	**87.9%**	**7.41 [4.22, 10.60]**
Heterogeneity: $Tau^2 = 0.00$; $Chi^2 = 2.75$, df = 3 (P = 0.43); $I^2 = 0\%$								
Test for overall effect: Z = 4.55 (P < 0.00001)								
**10.2.2 Severe**								
Cantillo-Negrete J 2021	23.1	16.1	10	21.9	15.5	10	4.9%	1.20 [-12.65, 15.05]
Fu J 2023	31.25	22.77	30	31.67	22.5	31	7.2%	-0.42 [-11.78, 10.94]
**Subtotal (95% CI)**			**40**			**41**	**12.1%**	**0.23 [-8.55, 9.02]**
Heterogeneity: $Tau^2 = 0.00$; $Chi^2 = 0.03$, df = 1 (P = 0.86); $I^2 = 0\%$								
Test for overall effect: Z = 0.05 (P = 0.96)								
**Total (95% CI)**			**93**			**90**	**100.0%**	**6.55 [3.49, 9.61]**
Heterogeneity: $Tau^2 = 0.20$; $Chi^2 = 5.04$, df = 5 (P = 0.41); $I^2 = 1\%$								
Test for overall effect: Z = 4.19 (P < 0.0001)								
Test for subgroup differences: $Chi^2 = 2.27$, df = 1 (P = 0.13), $I^2 = 55.9\%$								

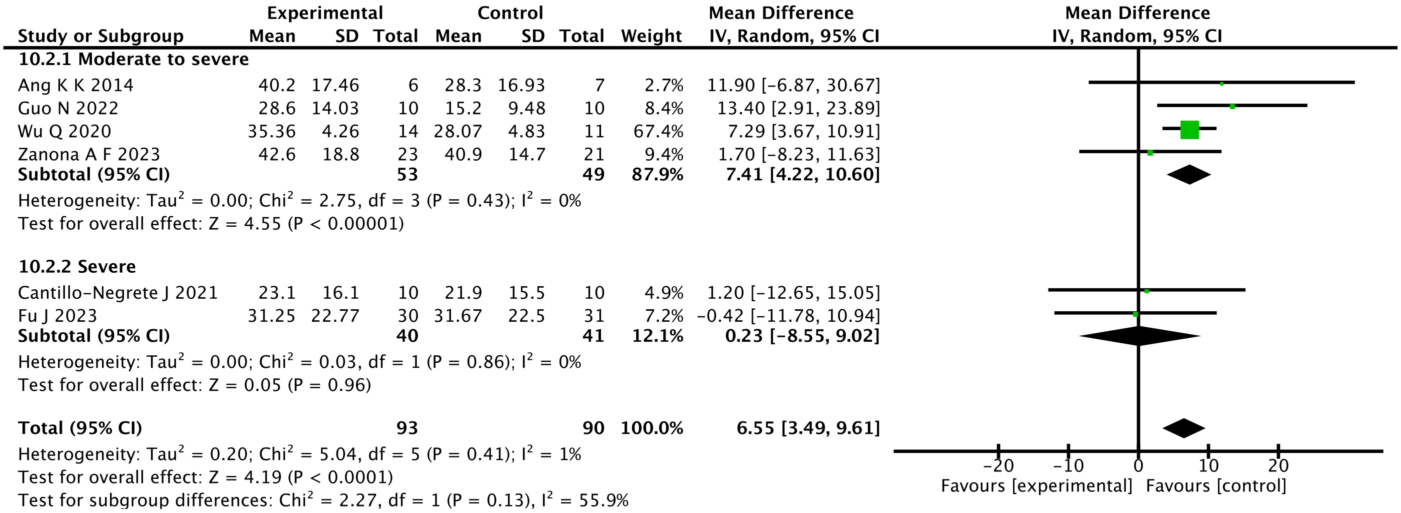

Supplementary Figure S19. Forest plot of subgroup analysis by upper limb impairment severity for FMA-UE: BCI-robot vs. conventional rehabilitation.

Study or Subgroup	Experimental Mean	Experimental SD	Experimental Total	Control Mean	Control SD	Control Total	Weight	Mean Difference IV, Random, 95% CI
**10.3.1 Moderate to severe**								
Cantillo-Negrete J 2025	21	24.94	10	19.43	33.5	9	4.8%	1.57 [-25.22, 28.36]
Cheng N 2020	5.4	4.8	5	9	8.85	5	44.4%	-3.60 [-12.42, 5.22]
Ji X 2025	20.58	19.75	20	12.67	12.01	19	33.2%	7.91 [-2.29, 18.11]
**Subtotal (95% CI)**			**35**			**33**	**82.4%**	**1.65 [-6.65, 9.96]**
Heterogeneity: $Tau^2 = 16.00$; $Chi^2 = 2.80$, df = 2 (P = 0.25); $I^2 = 28\%$								
Test for overall effect: Z = 0.39 (P = 0.70)								
**10.3.2 Severe**								
Frolov A A 2017	16.67	31.97	55	12.33	24.83	19	17.6%	4.34 [-9.66, 18.34]
**Subtotal (95% CI)**			**55**			**19**	**17.6%**	**4.34 [-9.66, 18.34]**
Heterogeneity: Not applicable								
Test for overall effect: Z = 0.61 (P = 0.54)								
**Total (95% CI)**			**90**			**52**	**100.0%**	**1.87 [-4.01, 7.75]**
Heterogeneity: $Tau^2 = 0.00$; $Chi^2 = 2.94$, df = 3 (P = 0.40); $I^2 = 0\%$								
Test for overall effect: Z = 0.62 (P = 0.53)								
Test for subgroup differences: $Chi^2 = 0.10$, df = 1 (P = 0.75), $I^2 = 0\%$								

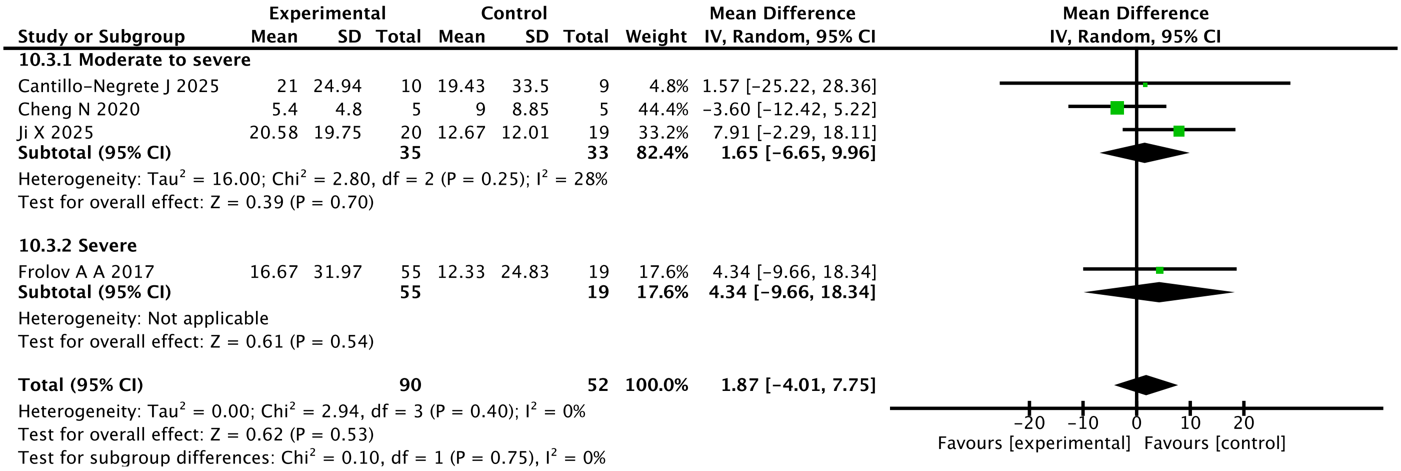

Supplementary Figure S20. Forest plot of subgroup analysis by upper limb impairment severity for ARAT: BCI-robot vs. robot.

Study or Subgroup	Experimental Mean	Experimental SD	Experimental Total	Control Mean	Control SD	Control Total	Weight	Mean Difference IV, Random, 95% CI
**11.1.1 6-week**								
Ang K K 2014	41.2	17.83	6	32	14.22	8	10.8%	9.20 [-8.14, 26.54]
Cheng N 2020	29	9.42	5	32.8	10.21	5	21.8%	-3.80 [-15.98, 8.38]
**Subtotal (95% CI)**			**11**			**13**	**32.6%**	**1.17 [-11.21, 13.56]**
Heterogeneity: $Tau^2 = 26.07$; $Chi^2 = 1.45$, df = 1 (P = 0.23); $I^2 = 31\%$								
Test for overall effect: Z = 0.19 (P = 0.85)								
**11.1.2 12-week**								
Guo N 2022	30.8	14.31	10	23.55	7.76	10	31.8%	7.25 [-2.84, 17.34]
**Subtotal (95% CI)**			**10**			**10**	**31.8%**	**7.25 [-2.84, 17.34]**
Heterogeneity: Not applicable								
Test for overall effect: Z = 1.41 (P = 0.16)								
**11.1.3 18-week**								
Ang K K 2014	42.7	17.83	6	33.8	14.65	8	10.5%	8.90 [-8.61, 26.41]
Cantillo-Negrete J 2025	32.67	16.34	10	33.6	30.79	9	6.4%	-0.93 [-23.45, 21.59]
Cheng N 2020	28.8	10.26	5	32	10.93	5	18.7%	-3.20 [-16.34, 9.94]
**Subtotal (95% CI)**			**21**			**22**	**35.7%**	**0.79 [-8.74, 10.31]**
Heterogeneity: $Tau^2 = 0.00$; $Chi^2 = 1.20$, df = 2 (P = 0.55); $I^2 = 0\%$								
Test for overall effect: Z = 0.16 (P = 0.87)								
**Total (95% CI)**			**42**			**45**	**100.0%**	**2.74 [-2.94, 8.43]**
Heterogeneity: $Tau^2 = 0.00$; $Chi^2 = 3.77$, df = 5 (P = 0.58); $I^2 = 0\%$								
Test for overall effect: Z = 0.95 (P = 0.34)								
Test for subgroup differences: $Chi^2 = 0.97$, df = 2 (P = 0.62), $I^2 = 0\%$								

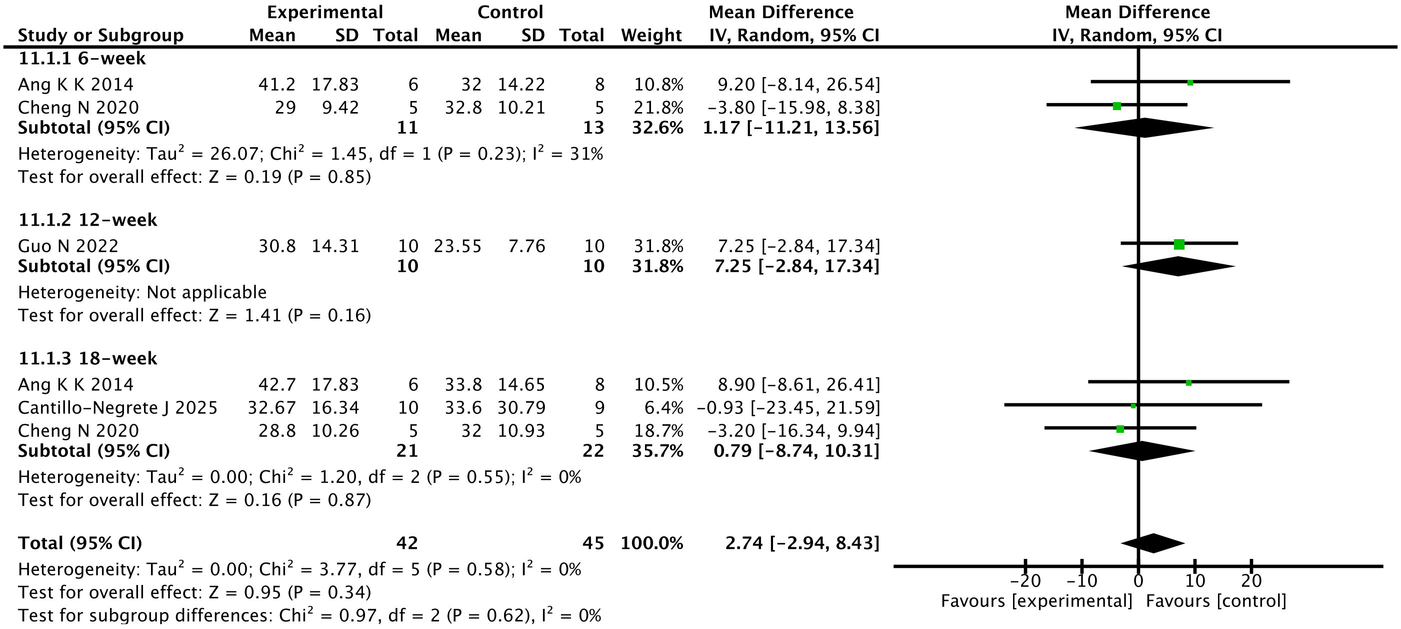

Supplementary Figure S21. Forest plot of subgroup analysis by follow-up duration for FMA-UE: BCI-robot vs. robot.

Study or Subgroup	Experimental Mean	Experimental SD	Experimental Total	Control Mean	Control SD	Control Total	Weight	Mean Difference IV, Random, 95% CI
**11.2.1 6-week**								
Ang K K 2014	41.2	17.83	6	27	17.9	7	18.6%	14.20 [-5.28, 33.68]
**Subtotal (95% CI)**			**6**			**7**	**18.6%**	**14.20 [-5.28, 33.68]**
Heterogeneity: Not applicable								
Test for overall effect: Z = 1.43 (P = 0.15)								
**11.2.2 12-week**								
Guo N 2022	30.8	14.31	10	16.3	9.24	10	63.2%	14.50 [3.94, 25.06]
**Subtotal (95% CI)**			**10**			**10**	**63.2%**	**14.50 [3.94, 25.06]**
Heterogeneity: Not applicable								
Test for overall effect: Z = 2.69 (P = 0.007)								
**11.2.3 18-week**								
Ang K K 2014	42.7	17.83	6	27	18.18	7	18.3%	15.70 [-3.92, 35.32]
**Subtotal (95% CI)**			**6**			**7**	**18.3%**	**15.70 [-3.92, 35.32]**
Heterogeneity: Not applicable								
Test for overall effect: Z = 1.57 (P = 0.12)								
**Total (95% CI)**			**22**			**24**	**100.0%**	**14.66 [6.27, 23.05]**
Heterogeneity: $Tau^2 = 0.00$; $Chi^2 = 0.01$, df = 2 (P = 0.99); $I^2 = 0\%$								
Test for overall effect: Z = 3.43 (P = 0.0006)								
Test for subgroup differences: $Chi^2 = 0.01$, df = 2 (P = 0.99), $I^2 = 0\%$								

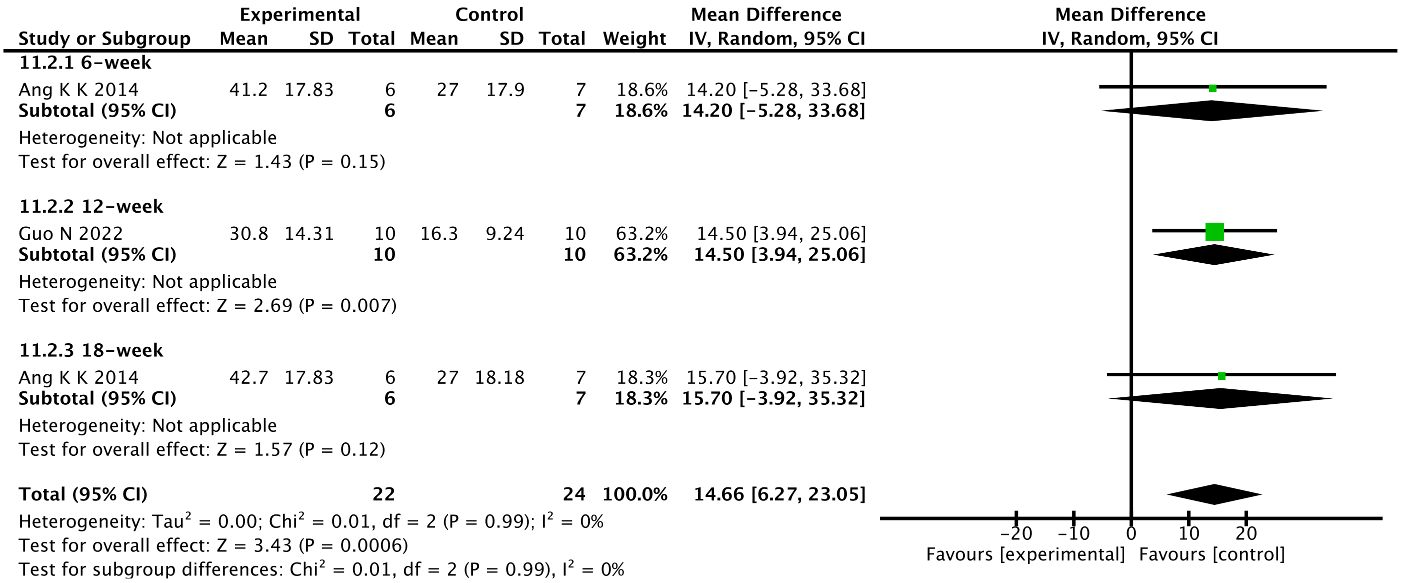

Supplementary Figure S22. Forest plot of subgroup analysis by follow-up duration for FMA-UE: BCI-robot vs. conventional rehabilitation.

Study or Subgroup	Experimental Mean	Experimental SD	Experimental Total	Control Mean	Control SD	Control Total	Weight	Mean Difference IV, Random, 95% CI
**11.3.1 6-week**								
Cheng N 2020	7.2	5.22	5	10	8.29	5	49.4%	-2.80 [-11.39, 5.79]
**Subtotal (95% CI)**			**5**			**5**	**49.4%**	**-2.80 [-11.39, 5.79]**
Heterogeneity: Not applicable								
Test for overall effect: Z = 0.64 (P = 0.52)								
**11.3.2 18-week**								
Cantillo-Negrete J 2025	19.43	32.94	10	23.93	39.19	9	3.4%	-4.50 [-37.25, 28.25]
Cheng N 2020	8.4	5.46	5	9	8.4	5	47.2%	-0.60 [-9.38, 8.18]
**Subtotal (95% CI)**			**15**			**14**	**50.6%**	**-0.86 [-9.34, 7.62]**
Heterogeneity: $Tau^2 = 0.00$; $Chi^2 = 0.05$, df = 1 (P = 0.82); $I^2 = 0\%$								
Test for overall effect: Z = 0.20 (P = 0.84)								
**Total (95% CI)**			**20**			**19**	**100.0%**	**-1.82 [-7.85, 4.22]**
Heterogeneity: $Tau^2 = 0.00$; $Chi^2 = 0.15$, df = 2 (P = 0.93); $I^2 = 0\%$								
Test for overall effect: Z = 0.59 (P = 0.55)								
Test for subgroup differences: $Chi^2 = 0.10$, df = 1 (P = 0.75), $I^2 = 0\%$								

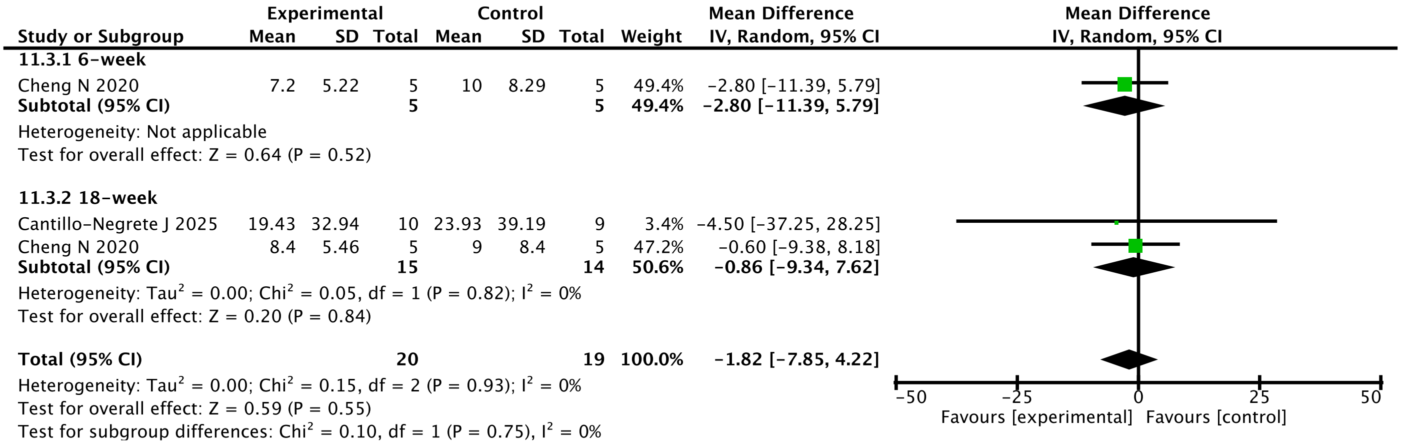

Supplementary Figure S23. Forest plot of subgroup analysis by follow-up duration for ARAT: BCI-robot vs. robot.
